# Structures of Fission Yeast Inositol Pyrophosphate Kinase Asp1 in Ligand-Free, Substrate-Bound, and Product-Bound States

**DOI:** 10.1128/mbio.03087-22

**Published:** 2022-12-05

**Authors:** Bradley Benjamin, Yehuda Goldgur, Nikolaus Jork, Henning J. Jessen, Beate Schwer, Stewart Shuman

**Affiliations:** a Molecular Biology Program, Memorial Sloan Kettering Cancer Center, New York, New York, USA; b Gerstner Sloan Kettering Graduate School of Biomedical Sciences, New York, New York, USA; c Structural Biology Program, Memorial Sloan Kettering Cancer Center, New York, New York, USA; d Institute of Organic Chemistry, University of Freiburg, Freiburg, Germany; e Department of Microbiology and Immunology, Weill Cornell Medical College, New York, New York, USA; Harvard Medical School

**Keywords:** Asp1 kinase, NTP donor specificity, *Schizosaccharomyces pombe*, inositol pyrophosphates, substrate binding

## Abstract

Expression of the fission yeast Schizosaccharomyces pombe phosphate regulon is sensitive to the intracellular level of the inositol pyrophosphate signaling molecule 1,5-IP_8_. IP_8_ dynamics are determined by Asp1, a bifunctional enzyme consisting of an N-terminal kinase domain and a C-terminal pyrophosphatase domain that catalyze IP_8_ synthesis and catabolism, respectively. Here, we report structures of the Asp1 kinase domain, crystallized with two protomers in the asymmetric unit, one of which was complexed with ligands (ADPNP, ADP, or ATP; Mg^2+^ or Mn^2+^; IP_6_, 5-IP_7_, or 1,5-IP_8_) and the other which was ligand-free. The ligand-free enzyme adopts an “open” conformation that allows ingress of substrates and egress of products. ADPNP, ADP, and ATP and associated metal ions occupy a deep phospho-donor pocket in the active site. IP_6_ or 5-IP_7_ engagement above the nucleotide favors adoption of a “closed” conformation, in which surface protein segments undergo movement and a disordered-to-ordered transition to form an inositol polyphosphate-binding site. In a structure mimetic of the kinase Michaelis complex, the anionic 5-IP_7_ phosphates are encaged by an ensemble of nine cationic amino acids: Lys43, Arg223, Lys224, Lys260, Arg274, Arg285, Lys290, Arg293, and Lys341. Alanine mutagenesis of amino acids that contact the adenosine nucleoside of the ATP donor underscored the contributions of Asp258 interaction with the ribose 3′-OH and of Glu248 with adenine-*N*^6^. Changing Glu248 to Gln elicited a gain of function whereby the kinase became adept at using GTP as phosphate donor. Wild-type Asp1 kinase can utilize *N*^6^-benzyl-ATP as phosphate donor.

## INTRODUCTION

Inositol pyrophosphate 1,5-IP_8_ is a signaling molecule that governs expression of the fission yeast Schizosaccharomyces pombe phosphate homeostasis (*PHO*) regulon (1) comprising three phosphate acquisition genes, *pho1*, *pho84*, and *tgp1*, that are repressed under phosphate-replete conditions by long noncoding RNA (lncRNA)-mediated transcriptional interference (2, 3). IP_8_ is generated from phytic acid (IP_6_) by the sequential action of fission yeast kinases Kcs1, which converts IP_6_ to 5-IP_7_, and Asp1, which converts 5-IP_7_ to 1,5-IP_8_ (4, 5). The *PHO* genes are dysregulated by genetic manipulations of Asp1, a bifunctional kinase and pyrophosphatase enzyme that consists of an N-terminal kinase domain that synthesizes 1,5-IP_8_ and a C-terminal pyrophosphatase domain that converts IP_8_ back to 5-IP_7_ (6–8). A pyrophosphatase-defective *asp1-H397A* allele (with a His-to-Ala change at residue 397) that increases the intracellular level of IP_8_ (6, 7) derepresses *pho1* expression by favoring precocious 3′-processing and termination of flanking *prt* lncRNA synthesis in response to poly(A) signals upstream of the mRNA promoter, in a manner dependent on the cleavage and polyadenylation factor (CPF) complex and transcription termination factor Rhn1 (1). An *asp1*Δ null allele, or a kinase-defective *D333A* allele, eliminates intracellular IP_8_ and results in *pho1* hyperrepression. Synthetic lethality of *asp1*Δ (no IP_8_) with CPF subunit mutations suggests that IP_8_ plays an important agonist role in essential 3′-processing and termination events, albeit in a manner genetically redundant to CPF (1).

Asp1 orthologs Vip1 (budding yeast), PPIP5K1 and PPIP5K2 (human paralogs), and VIH1 and VIH2 (plant paralogs) have been characterized genetically and biochemically. Studies in fungi have established that IP_8_ is inessential, insofar as Saccharomyces cerevisiae *vip1*Δ, Schizosaccharomyces pombe *asp1*Δ, and Cryptococcus neoformans *asp1*Δ null strains are viable and fit (1, 6, 9, 10). Human HCT116 cells were viable after CRISPR-mediated simultaneous disruption of the *IP6K1* and *IP6K2* genes encoding two Kcs1/IP6K enzyme paralogs, a maneuver that eliminated both IP_7_ and IP_8_ (11). Whereas single knockouts of *Arabidopsis* VIH1 and VIH2 had no effect on plant growth, a double knockout impaired growth (12).

The isolated N-terminal kinase domains of Asp1, Vip1, PPIP5K, and VIH have autonomous kinase activity (6–8, 13–15). Wang et al. conducted incisive structural and mechanistic studies of the kinase domain of human PPIP5K2 (16). Their structures delineated the binding sites for the ATP and inositol polyphosphate substrates, captured a MgF_3_ mimetic of the kinase reaction transition state, and inspired the identification of essential active site constituents via mutagenesis (16). We recently reported a biochemical characterization of the isolated fission yeast Asp1 kinase domain (amino acids [aa] 1 to 385) and a mutational analysis of Asp1 guided by the PPIP5K2 structure (8). PPIP5K2 and Asp1 share several biochemical properties. In particular, the reaction of the PPIP5K2 and Asp1 kinases with ATP·Mg^2+^ and IP_6_ has two parallel outcomes—(i) formation of 1-IP_7_ and ADP after γ-phosphate transfer to IP_6_ and (ii) formation of ADP via γ-phosphate transfer to water—that comprise productive and unproductive modes of catalysis with respect to inositol pyrophosphate metabolism. Under reaction conditions optimal for IP_6_ kinase activity, the Asp1 reaction path is split almost equally between kinase and ATPase (8). Whereas ATP hydrolysis by Asp1 does not depend on IP_6_, the ATPase of human PPIP5K2 kinase is strongly stimulated by the presence of IP_6_ and other inositol polyphosphates (15–17). It has been proposed that occupancy of a second inositol polyphosphate-binding site (a substrate capture site) adjacent to and overlapping the IP_6_ phospho-acceptor site can stimulate ATP hydrolysis by PPIP5K2 (17, 18). It is not known whether Asp1 has an analogous preloading site for inositol polyphosphates. When 5-IP_7_ is provided as the substrate for Asp1 kinase, IP_8_ synthesis is favored by >30-fold over the hydrolysis of ATP, and the rate of phosphorylation of 5-IP_7_ by Asp1 kinase is 22-fold faster than the rate of IP_6_ phosphorylation (8). Similar findings have been reported for the human PPIP5K2 kinase domain, whereby the first-order rate constant for 5-IP_7_ phosphorylation is 22-fold greater than the rate constant for IP_6_ phosphorylation (16), and the *k*_cat_/*K_m_* for 5-IP_7_ phosphorylation is 29-fold greater than for IP_6_ phosphorylation (15). We found that the strong preference of Asp1 kinase for 5-IP_7_ versus IP_6_ as the phosphate acceptor was maintained under competitive substrate conditions in which IP_6_ was present in molar excess over 5-IP_7_ (8).

To gain further insight into Asp1, we aimed here to capture crystal structures of the Asp1 kinase domain in various states along its reaction pathways.

## RESULTS

### Trimming the margins of the Asp1 kinase domain.

Crystallization trials with the N-terminal kinase domain Asp1-(1-385), which we characterized biochemically (8), yielded crystals that diffracted X-rays poorly. Success was achieved after trimming 30 aa from the N terminus and truncating the C terminus at position 364. The purified recombinant Asp1-(31-364) protein (see Fig. S1A in the supplemental material), like its longer progenitor, was catalytically active in converting 5-IP_7_ to 1,5-IP_8_ in the presence of ATP and magnesium (Fig. S1B).

10.1128/mbio.03087-22.1 FIG S1 Trimming the margins of the Asp1 kinase domain. (A) Aliquots (5 μg) of the peak Superdex 200 fractions of the Asp1-(1-385) and Asp1-(31-364) kinase domain proteins were analyzed by SDS-PAGE. The Coomassie-blue stained gel is shown. The positions and sizes (in kilodaltons) of marker polypeptides are indicated on the left. (B) Kinase reaction mixtures (20 μL) containing 30 mM bis-Tris (pH 6.2), 50 mM NaCl, 5 mM MgCl_2_, 2 mM ATP, 0.5 mM 5-IP_7_, and 0.5 μM (10 pmol) Asp1-(1-385) or Asp1-(31-364), as specified, were incubated at 37°C for 15 min. Asp1 was omitted from a control reaction in lane –. The reactions were quenched by addition of 1 μL of 0.5 M EDTA. The mixtures were adjusted to 15% glycerol and 0.05% Orange G dye and analyzed by electrophoresis through a 35% polyacrylamide gel in TBE buffer (80 mM Tris-borate [pH 8.3], 1 mM EDTA) at 4°C (8 W constant power, for ~4 h). ATP and inositol pyrophosphates were visualized by staining the gel with toluidine blue. Download FIG S1, JPG file, 0.1 MB. Copyright © 2022 Benjamin et al. 2022 Benjamin et al. https://creativecommons.org/licenses/by/4.0/ This content is distributed under the terms of the Creative Commons Attribution 4.0 International license.

### Crystallization of Asp1 kinase.

A solution of 0.1 mM Asp1-(31-364), 1 mM ADPNP, 5 mM Mg^2+^, and 1 mM IP_6_ was mixed with an equal volume of precipitant solution containing 0.1 M bis-Tris (pH 5.5), 0.1 M NH_4_-acetate [NH_4_OAc], 17% polyethylene glycol 10,000 (PEG 10,000). Crystals were grown by sitting-drop vapor diffusion at room temperature. The Asp1 kinase crystals diffracted X rays to 1.9 Å resolution, were in space group P2_1_, and contained two Asp1 kinase protomers in the asymmetric unit. The structure was solved as described in Materials and Methods and summarized in Table S1 (R_work_/R_free_ = 0.197/0.226). The A protomer comprised a continuous polypeptide from Asn33 to Glu362; the B protomer was punctuated by a disordered 20-aa segment (aa 275 to 294) for which no interpretable electron density was evident. The A protomer contained ADPNP and IP_6_ in the active site and was taken to exemplify an on-pathway bisubstrate complex. The B protomer was a ligand-free enzyme.

10.1128/mbio.03087-22.6 TABLE S1 Crystallographic data and refinement statistics (data in parentheses refer to the highest-resolution shell). Download Table S1, PDF file, 0.04 MB. Copyright © 2022 Benjamin et al. 2022 Benjamin et al. https://creativecommons.org/licenses/by/4.0/ This content is distributed under the terms of the Creative Commons Attribution 4.0 International license.

### Overview of the kinase structure.

**FIG 1:**
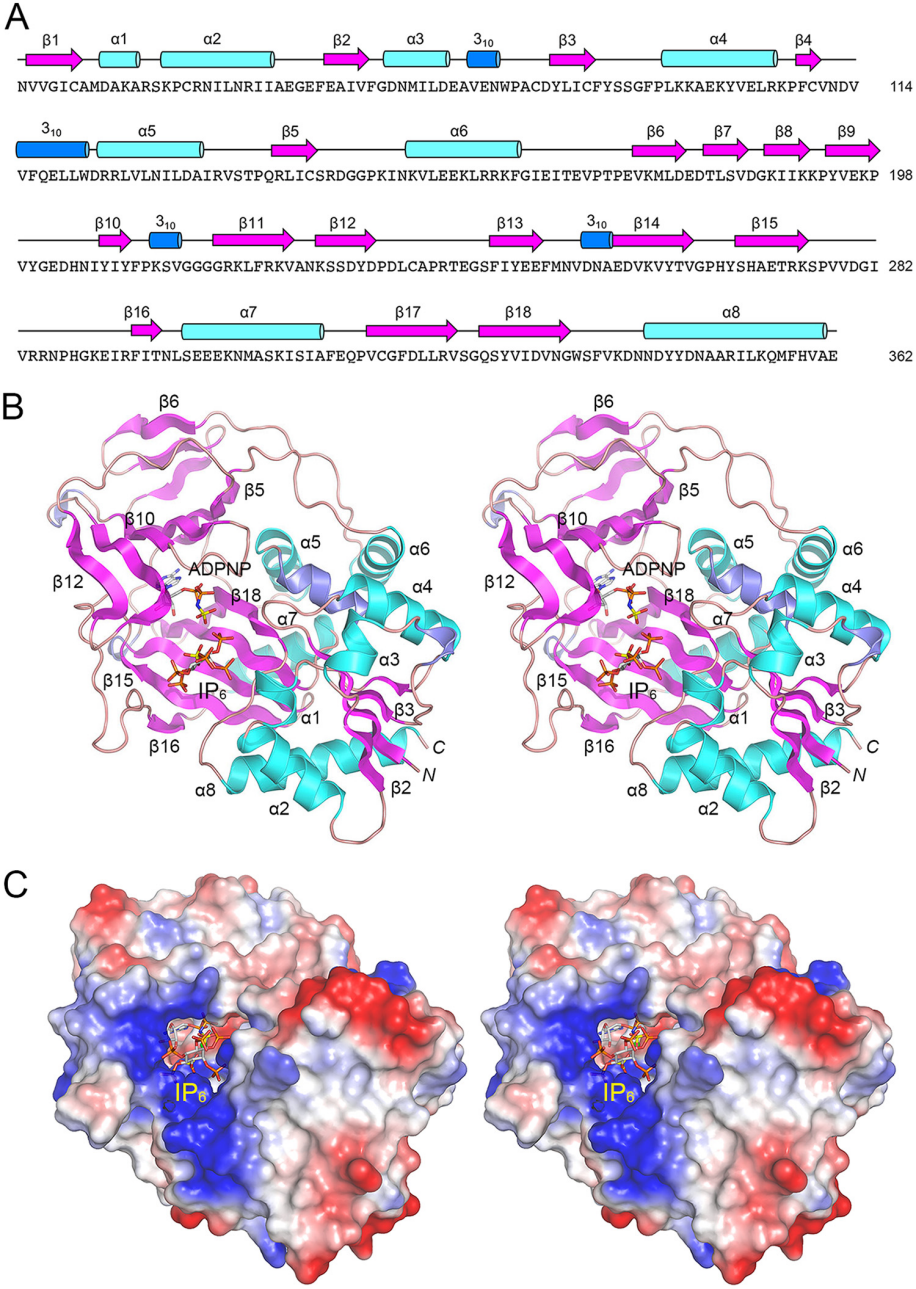
Structure of Asp1 kinase. (A) The amino acid sequence of Asp1 from Asn33 to Glu362 is shown. The secondary structure elements are displayed above the primary structure, with β-strands depicted as magenta arrows, α-helices as cyan cylinders, and 3_10_ helices as blue cylinders. (B) Stereoview of the tertiary structure of the A protomer, depicted as a cartoon model with magenta β-strands, cyan α-helices, and blue 3_10_ helices. The secondary structure elements are labeled according to their order in the primary structure, as for panel A. ADPNP and IP_6_ are rendered as stick models with gray carbons. The ADPNP γ-phosphorus and IP_6_ 1-phosphorus atoms are colored yellow. (C) Stereoview of a surface electrostatic model of the A protomer generated in PyMOL, looking down at the active site pocket, with IP6 (stick model) exposed to solvent at the rim of the active site, where it is flanked by positive electrostatic potential (blue).

**FIG 2:**
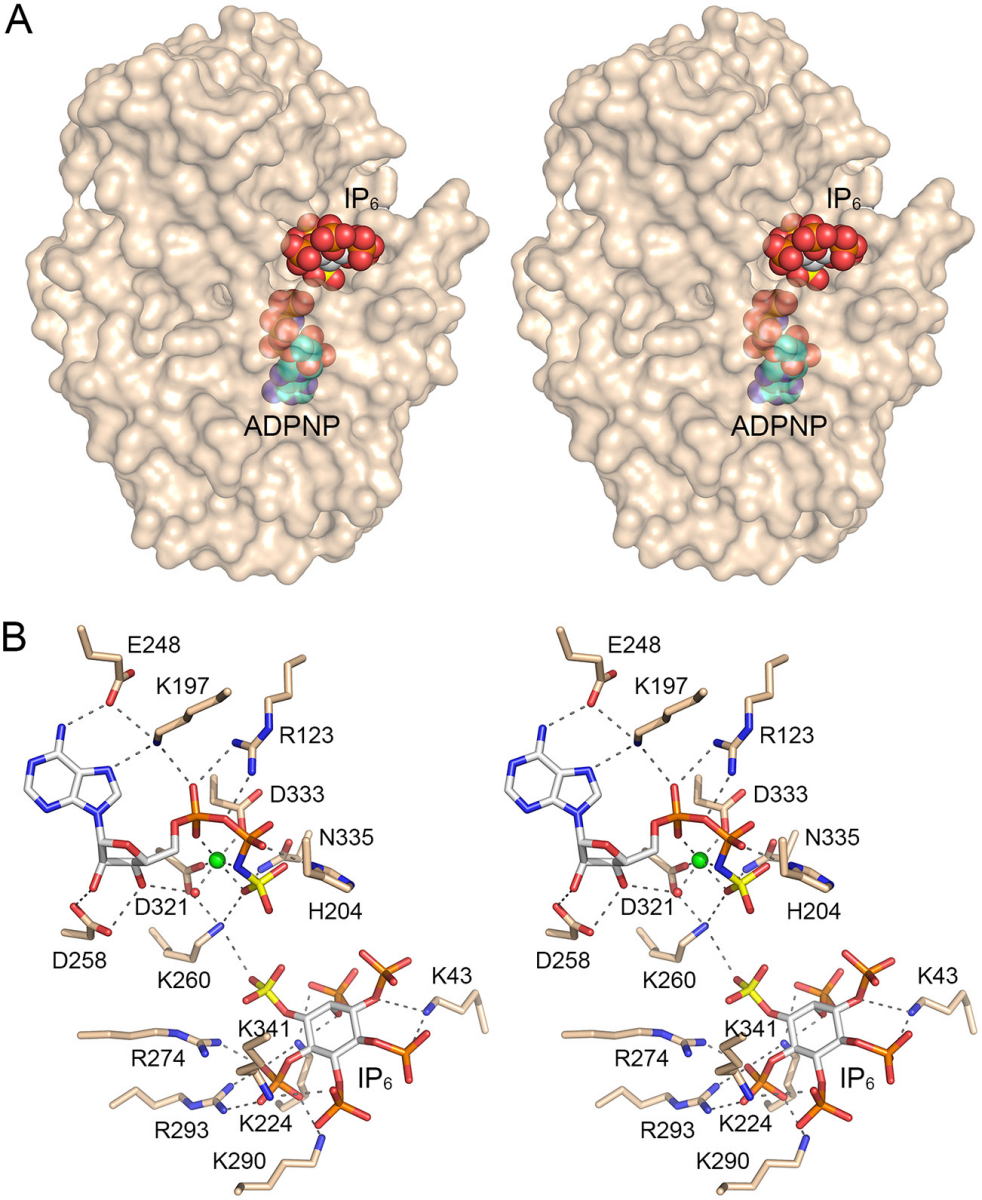
Asp1 kinase in complex with ADPNP and IP_6_. (A) Stereoview of a semitransparent surface model of the A protomer with ADPNP (cyan carbons), IP_6_ (gray carbons; yellow for 1-phophorus), and magnesium (green) atoms rendered as spheres. (B) Stereoview of the active site of the A protomer with selected amino acids depicted as stick models with beige carbons. ADPNP and IP_6_ are rendered as stick models with gray carbons. The ADPNP γ-phosphorus and IP_6_ 1-phosphorus atoms are colored yellow. Magnesium and a water in the octahedral metal coordination complex are depicted as green and red spheres, respectively. Atomic contacts are indicated by dashed lines.

A stereoview of the A protomer tertiary structure is shown in Fig. 1B. It is composed of 18 β-strands, eight α-helices, and four 3_10_ helices, which are displayed over the amino acid sequence in Fig. 1A. ADPNP, rendered as a stick model in Fig. 1B, is bound within a pocket lined by a 6-strand antiparallel β-sheet (with topology β12↑•β1↓•β10↑•β9↓•β13↑•β5↓) and a 5-strand antiparallel β-sheet (β16↓•β15↑•β14↓•β17↑•β18↓). A 3-strand antiparallel sheet (β6↑•β7↓•β8↑) forms a β-sandwich over the 6-strand sheet. An N-terminal 4-strand parallel β-sheet (β2↑•β1↑•β3↑•β4↑) is flanked on both sides by helices. A surface electrostatic model (Fig. 1C) and a semitransparent surface model with ligand atoms rendered as spheres (Fig. 2A) highlighted that ADPNP is buried within a deep active site pocket, whereas IP_6_ is exposed to solvent at the rim of the active site, where it is surrounded by positive electrostatic potential conducive to engagement of the anionic phosphate groups. Because IP_6_ occupancy effectively occludes the mouth of the active site (Fig. 2A), it must be the case that Asp1 kinase adheres to an ordered bi-bi mechanism in which ATP binds to the enzyme before binding of the IP_6_ or 5-IP_7_ phospho-acceptor substrate and the 1-IP_7_ or 1,5-IP_8_ product dissociates prior to the release of ADP.

**FIG 3:**
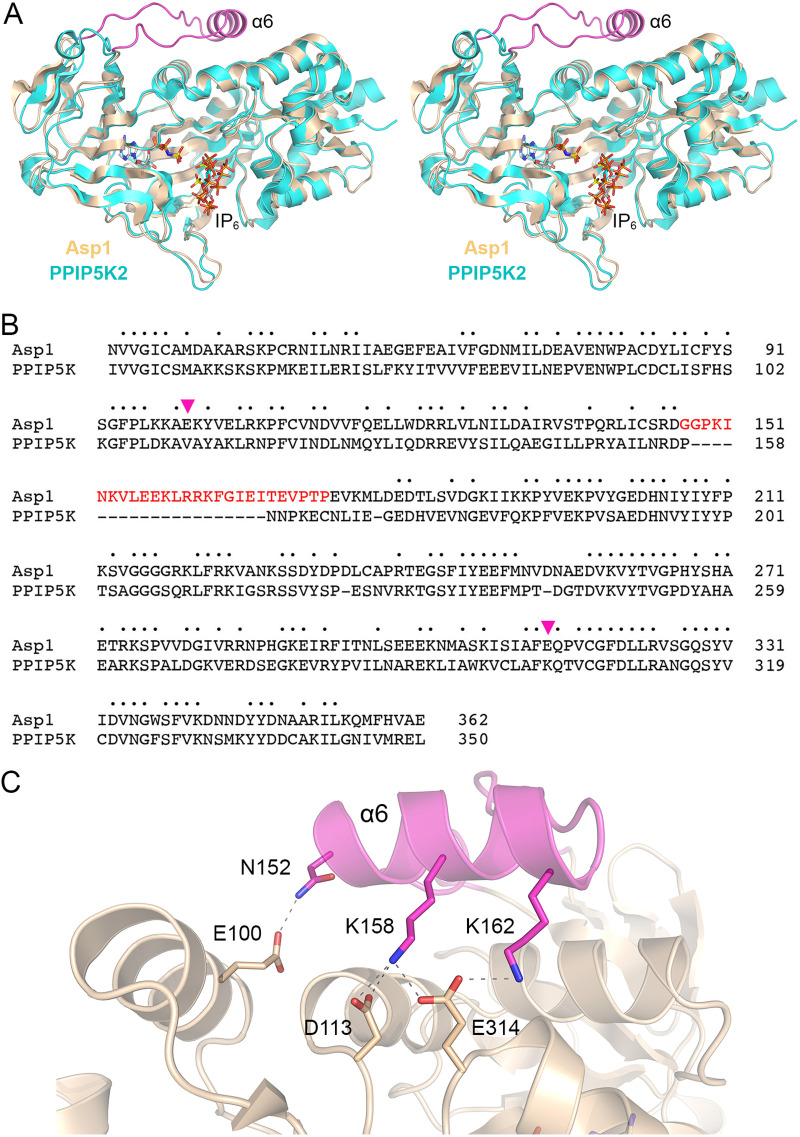
Structural comparison of Asp1 and PPIP5K2. (A) Stereoview of the superimposed crystal structures of Asp1 kinase in complex with ADPNP and IP_6_ (gold cartoon model) and human PPIP5K2 in complex with ADPNP and IP_6_ (cyan cartoon model) (PDB 3T9C). An internal Asp1 segment containing the α6-helix that has no counterpart in PPIP5K2 is colored magenta. (B) The amino acid sequences of the fission yeast Asp1 and human PPIP5K2 kinase domains are aligned. Positions of side chain identity are denoted by dots above the Asp1 sequence. The Asp1 segment from Gly147 to Pro173, which has no counterpart in PPIP5K2, is highlighted in red. (C) View of the Asp1 tertiary structure highlighting intramolecular contacts of α6 amino acids Asn152, Lys158, and Lys162 with acidic residues Glu100, Asp113, and Glu314. Glu100 and Glu314 are indicated by magenta arrowheads in panel B and are not conserved in PPIP5K2.

A DALI search (19) of the PDB recovered multiple members of the ATP-grasp protein family (20) as structural homologs of Asp1 kinase (Table S2). The top “hit” was the human ortholog PPIP5K2 (*Z* score of 41.1, 51% amino acid identity and root mean square deviation of 1.4 Å at 304 Cα positions) (16). PPIP5K2 catalyzes the same kinase reaction as Asp1. Lower homology (with *Z* scores between 21 and 17) was apparent to the ATP-grasp proteins Escherichia coli glutamate ligase RimK (21), human inositol tetrakisphosphate 1-kinase (22), *Entamoeba* inositol trisphosphate 5/6-kinase (23), *Streptomyces* cycloserine biosynthesis protein DcsG (24), rat synapsin (25), macrocyclase PsnB (26), and d-alanine-d-alanine ligase (27). DALI-based alignments of the fission yeast Asp1 and human PPIP5K2 tertiary and primary structures are shown in Fig. 3A and B, respectively. The salient point is that Asp1 has a 27-aa structural element inserted between β5 and β6 (containing the Asp1 α6-helix) that is not present in PPIP5K2; nor is this Asp1 element present in any of the other top DALI hits cited above. Although the function of this Asp1-specific insert is unclear, three amino acids within α6 (Asn152, Lys158, and Lys162) make hydrogen bonds or salt bridges to acidic amino acids (Glu100, Asp113, and Glu314) of the main body of the protein (Fig. 3C). Two of the recipient amino acids (Glu100 and Glu314) are not conserved in PPIP5K2 (Fig. 3B).

10.1128/mbio.03087-22.7 TABLE S2 Closest structural homologs of S. pombe Asp1 kinase identified by DALI. Download Table S2, PDF file, 0.02 MB. Copyright © 2022 Benjamin et al. 2022 Benjamin et al. https://creativecommons.org/licenses/by/4.0/ This content is distributed under the terms of the Creative Commons Attribution 4.0 International license.

### The kinase active site in complex with ADPNP and IP_6_.

Figure 2B shows a stereoview of the active site of the A protomer, highlighting Asp1 enzymatic interactions with ADPNP·Mg^2+^ and IP_6_. Asp1 prefers ATP as the phosphate donor in the IP_6_ kinase reaction; other standard ribonucleoside triphosphates are either ineffective (UTP) or weakly effective (GTP and CTP) (8). Adenine preference is apparently enforced by a hydrogen bond from Glu248 to the adenine-*N*^6^ atom. Lys197 makes bifurcated interaction with adenine-*N*^7^ and the ATP α-phosphate. Asp258 makes bidentate hydrogen bonds to the ATP ribose 2′-OH and 3′-OH groups. The conformation of the triphosphate moiety is stabilized by an octahedrally coordinated magnesium ion. The six sites in the metal complex are occupied by α- and γ-phosphate oxygens, the β-γ-bridging nitrogen of ADPNP, the Asp321-Oδ2 and Asp333-Oδ2 atoms, and a water that bridges to the ribose 3′-OH (Fig. 2B). Additional contacts to the ATP phosphates include the following: a bidentate interaction of Arg123 with α-phosphate and α-β-bridging phosphate oxygens; a hydrogen bond from His204-Nδ to the β-phosphate; a hydrogen bond from Asn335-Nδ to the γ-phosphate; and a bifurcated interaction of Lys260 with the γ-phosphate and 1-phosphate of IP_6_ (Fig. 2B). Previous alanine-scanning mutagenesis had established that metal ligands Asp321 and Asp333, and also ATP phosphate ligands His204 and Lys260, were essential for Asp1’s 5-IP_7_ kinase activity, whereas ATP phosphate ligands Asn335 and Arg123 were not essential (8). Superposition of ADPNP-bound Asp1 on ADPNP-bound PPIP5K2 showed that the nucleotide positions and conformations were virtually identical (Fig. 3A). One difference was that the PPIP5K2 ADPNP complex included two magnesium ions engaged to the nucleotide (16), whereas our initial Asp1 structure had one magnesium ion. By including a metal ion during cryoprotection, or growing crystals in the presence of manganese, we were subsequently able to capture Asp1 with two metals in the active site (see below).

IP_6_ is depicted in Fig. 2 with a yellow phosphorus atom at its 1-phosphate group, an oxygen of which is the nucleophile that attacks the ATP γ-phosphorus (also colored yellow). An IP_6_ 1-phosphate oxygen is positioned 4.8 Å from the γ-phosphorus and is oriented relative to the ADP leaving group (exemplified in the structure by the β-γ-bridging nitrogen) at an O-Pγ-N angle of 143°, signifying that the distance must close and the angle increase as the substrate complex progresses toward a transition state for phosphoryl transfer in which the nucleophile is apical to the leaving group. We envision that the essential bridging Lys260 residue is critical to overcome the charge repulsion of the reactive phosphate groups. Asp1 makes multiple electrostatic interactions with the five other phosphates of IP_6_, as follows: Arg293 makes a bidentate contact to the 6-phosphate; Lys290 and Arg274 contact the 6-phosphate; Lys224 engages the 5-phosphate; Lys341 makes bifurcated contacts to the 2- and 6-phosphates; Lys43 contacts the 3- and 4-phosphates (Fig. 2B). A ligand plot of the Asp1-IP_6_ interface, with atomic distances, is shown in Fig. S2. The superposition of the substrate-bound Asp1 and PPIP5K2 structures in Fig. 3A showed that, whereas the IP_6_ ligands were oriented almost identically with respect to the six phosphate groups, the IP_6_ in the Asp1 structure was shifted by ~2 Å compared to PPIP5K2 (i.e., the individual inositol carbons were offset by 1.6 to 2.3 Å in the two structures). This shift accounted for the longer distance from an IP_6_ 1-phosphate oxygen to the ADPNP γ-phosphorus in Asp1 (4.8 Å) versus PPIP5K2 (4.1 Å).

10.1128/mbio.03087-22.2 FIG S2 LIGPLOT schematic diagram of the enzymic contacts between Asp1 kinase and IP_6_. Download FIG S2, JPG file, 0.3 MB. Copyright © 2022 Benjamin et al. 2022 Benjamin et al. https://creativecommons.org/licenses/by/4.0/ This content is distributed under the terms of the Creative Commons Attribution 4.0 International license.

### Conformational differences in substrate-bound versus substrate-free Asp1.

**FIG 4:**
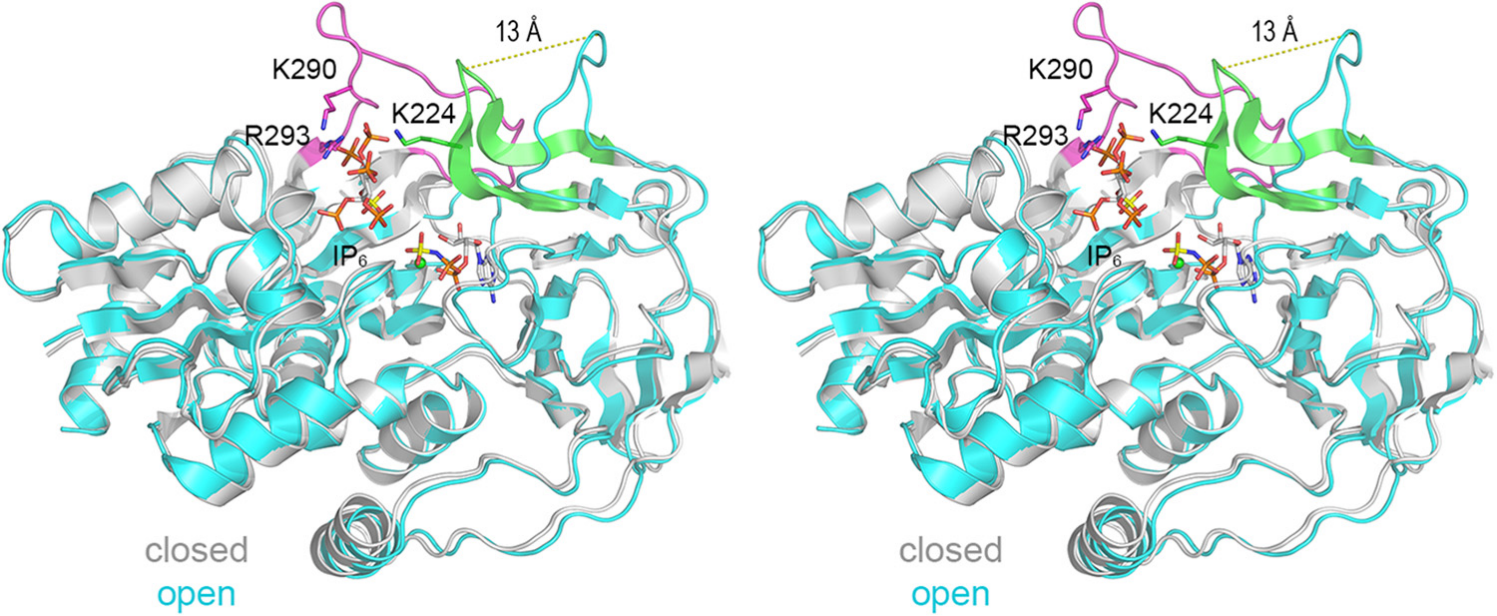
Conformational differences in substrate-bound versus substrate-free Asp1. Stereoview of the superimposed structures of the substrate-bound A protomer (closed conformation; gray cartoon model) and ligand-free B protomer (open conformation; cyan cartoon model). A disordered 20-aa segment (aa 275 to 294) of the B protomer that becomes ordered in the IP_6_-bound A protomer to form the β15-β16 loop is colored magenta. Side chains K224, K290, and R293 of the A protomer are depicted as stick models. A 13-aa segment from aa 220 to 232, which is a loop that has little secondary structure in the B protomer and is oriented away from the mouth of the active site, folds into well-defined β11 and β12 strands in the A protomer (colored green) and undergoes a 13-Å movement with respect to the Asn227 Cα (denoted by the dashed line).

Superposition of the structures of the substrate-bound A protomer (gray cartoon model in Fig. 4) and the ligand-free B protomer (cyan cartoon model in Fig. 4) revealed a conformational transition involving two elements involved in IP_6_ binding. First, the disordered 20-aa segment (aa 275 to 294) of the ligand-free enzyme becomes ordered in the IP_6_-bound state to form the β15-β16 loop (colored magenta in Fig. 4), which contributes to the positively charged rim of the active site pocket. Lys290 and Arg293 within this loop contact the phosphates of IP_6_ (Fig. 4). Second, the 13-aa segment from aa 220 to 232, which is a loop that has little secondary structure in the ligand-free enzyme and is oriented away from the mouth of the active site, folds into well-defined β11 and β12 strands in the A protomer and undergoes a 13-Å movement with respect to the Asn227 Cα (Fig. 4). This movement brings β11-β12 to the rim of the active site pocket and Lys224 into contact with IP_6_. (The β11-β12 segment in the A protomer makes a single lattice contact to the B subunit in the same asymmetric unit, entailing a hydrogen bond from Asn227-Nδ to a main-chain carbonyl of Ile57. In the ligand-free B protomer, Ser230-Oδ and the Asp231 main-chain amide donate hydrogen bonds to the Phe163 carbonyl of the A protomer in a neighboring asymmetric unit.) We surmise that the B protomer exemplifies an “open” conformation that allows ingress of the substrates and that IP_6_ (or 5-IP_7_) binding triggers the “closed” conformation seen in the A protomer that is on-pathway for catalysis. We presume that the reverse transition occurs after the kinase reaction to facilitate product dissociation.

### Structure of Asp1 in complex with ADPNP and 5-IP_7_.

**FIG 5:**
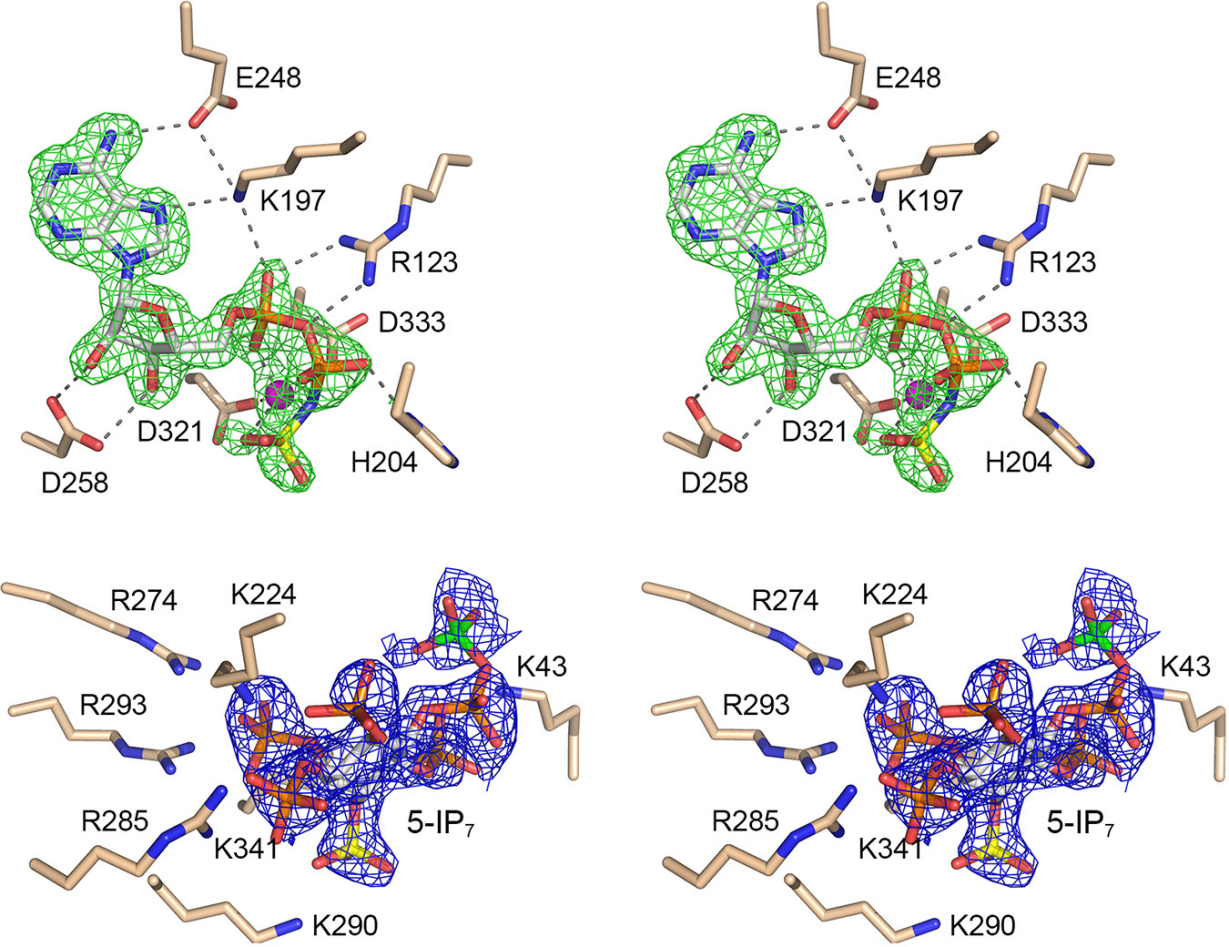
Asp1 kinase in complex with ADPNP and 5-IP_7_. Stereoview of the active site of the A protomer with selected amino acids depicted as stick models with beige carbons. ADPNP is rendered as a stick model with gray carbons and a yellow γ-phosphorus atom, adjacent to a magnesium ion (magenta sphere). 5-IP_7_ is a stick model with carbons in gray, the 1-phosphorus atom colored yellow, and the 5-β-phosphorus atom colored green. Omit density maps overlying the ADPNP and 5-IP_7_ molecules are contoured at 1σ and depicted as green and blue mesh, respectively. Atomic contacts of ADPNP and Mg^2+^ are indicated by dashed lines.

A crystal grown from a premixture of 0.1 mM Asp1-(31-364), 1 mM ADPNP, 5 mM Mg^2+^, and 1 mM 5-IP_7_ diffracted X rays to 1.7 Å resolution and was isomorphous with the crystal described above. The A protomer contained ADPNP and 5-IP_7_ in the active site. The B protomer was ligand-free. Figure 5 shows a stereoview of the active site, with omit density overlying the ADPNP and 5-IP_7_ molecules depicted as green and blue mesh, respectively. A single magnesium ion coordinates α- and γ-phosphate oxygens, the β-γ-bridging nitrogen of ADPNP, and the Asp321-Oδ2 and Asp333-Oδ2 atoms. The position and orientation of the 5-IP_7_ ligand (depicted with the 1-phosphorus colored yellow and the 5-β-phosphorus colored green) is off-pathway with respect to catalysis of the kinase reaction, notwithstanding that 5-IP_7_ is surrounded by the same cage of positively charged amino acids—Arg274, Arg293, Lys290, Lys224, Lys341, and Lys43—that engage IP_6_ in a more plausible on-pathway mode. In the IP_7_-bound complex, the 1-phosphate group is directed away from the ADPNP phosphate donor, at a distance of 15 Å from the γ-phosphorus. It is conceivable that this mode of inositol polyphosphate binding to Asp1 is analogous to the “capture site” described by Wang and colleagues for human PPIP5K2 (17).

### Structures of Asp1 in complex with nucleotide and two metal ions.

**FIG 6:**
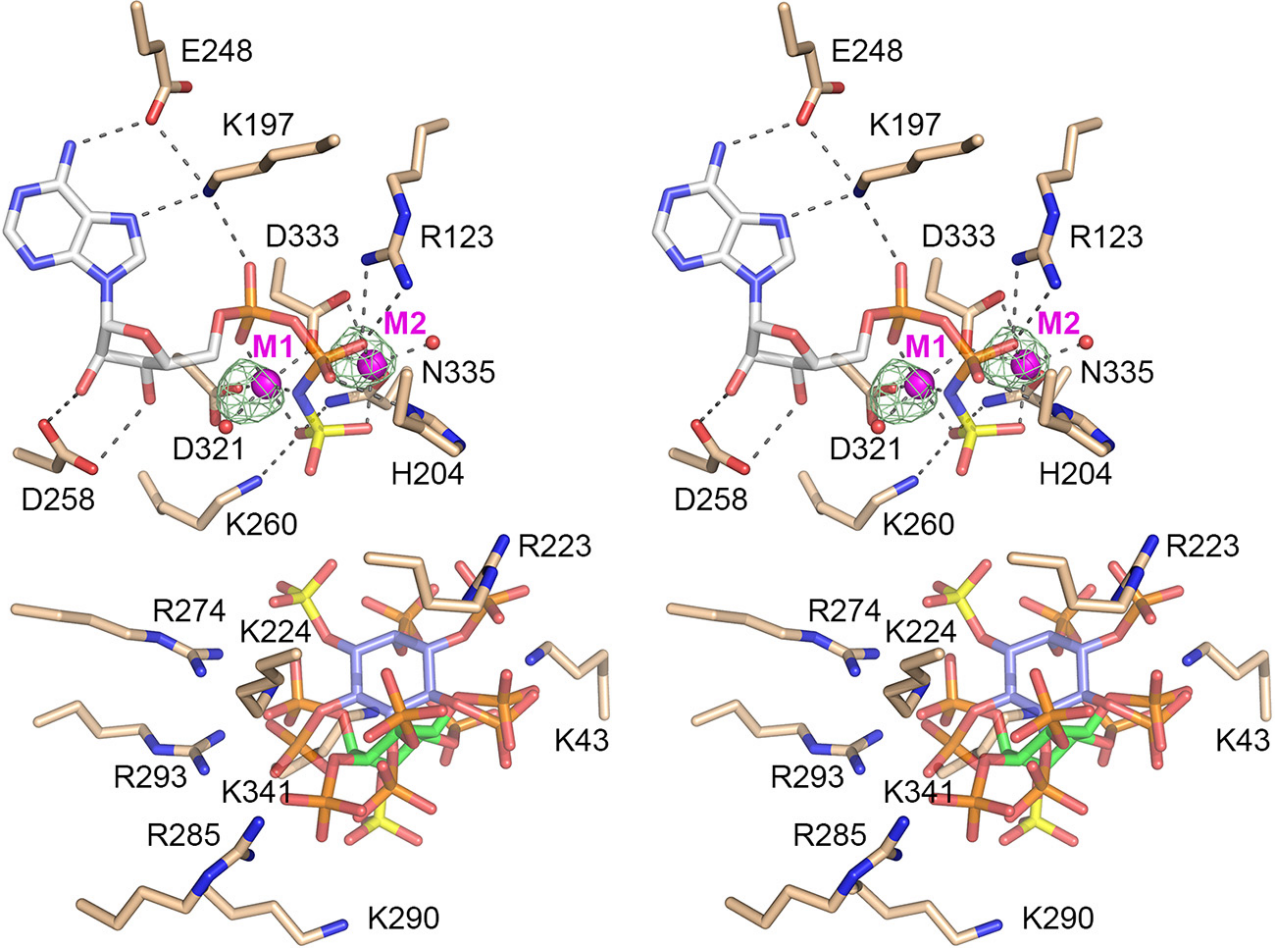
Asp1 kinase in complex with ADPNP, two manganese ions, and IP_6_. Stereoview of the active site of the A protomer with selected amino acids depicted as stick models with beige carbons. ADPNP is rendered as a stick model with gray carbons and a yellow γ-phosphorus atom, adjacent to two manganese ions (M1 and M2; magenta spheres). Metal-bound waters are red spheres. Two different orientations of IP_6_ (shown with either green or blue inositol carbon atoms and yellow 1-phosphorus atoms), each with partial occupancy, were modeled in the phospho-acceptor site. Atomic contacts of ADPNP, Mn1, and Mn2 are indicated by dashed lines. The anomalous difference peaks overlying the manganese atoms are depicted as a green mesh contoured at 4σ.

A crystal grown by seeding from a premixture of 0.1 mM Asp1-(31-364), 1 mM ADPNP, 5 mM Mn^2+^, and 1 mM IP_6_ diffracted X rays to 1.7 Å resolution and contained two Asp1 kinase protomers in the asymmetric unit, one being free enzyme and the other containing ADPNP and IP_6_. A stereoview of the active site of the substrate-bound protomer is shown in Fig. 6 and is notable for the presence of two metal ions adjacent to the ADPNP. Anomalous difference peaks were calculated from diffraction data collected for a second crystal grown under the same conditions, in which the unit cell dimensions differed from the first crystal by ~0.5 Å (a, b, c = 47.53, 87.19, 86.42 Å; α, β, γ = 90.00, 94.76, 90.00°). The anomalous signal was stronger in the second crystal than in the first crystal, while the density for the IP_6_ ligand was less well defined in the second crystal. The anomalous peaks overlying the manganese atoms are depicted as green mesh in Fig. 6, contoured at 4σ. The Mn^2+^ in the M1 site (equivalent to the Mg^2+^ seen in Fig. 2) is coordinated octahedrally to α- and γ-phosphate oxygens, the β-γ-bridging nitrogen of ADPNP, the Asp321-Oδ2 and Asp333-Oδ2 atoms, and a water. The Mn^2+^ in the M2 site is coordinated octahedrally to β- and γ-phosphate oxygens, the Asp333-Oδ2 and -Oδ1 atoms, Asn335-Oδ, and a water. The other enzymatic contacts to ADPNP·(Mn^2+^)_2_ are the same as in the ADPNP·Mg^2+^ structure. We modeled two different orientations of IP_6_ (depicted in Fig. 6 with either green or blue inositol carbon atoms), each with partial occupancy, to fit the electron density in the phospho-acceptor site. In one of the IP_6_ molecules, the nearest 1-phosphate oxygen is 4.9 Å from the ADPNP γ-phosphorus (similar to the IP_6_ seen in the ADPNP·Mg^2+^ structure) (Fig. 2). In the other IP_6_, the 1-phosphate is 15 Å away from the ADPNP γ-phosphate (akin to the 5-IP_7_ in Fig. 5).

**FIG 7:**
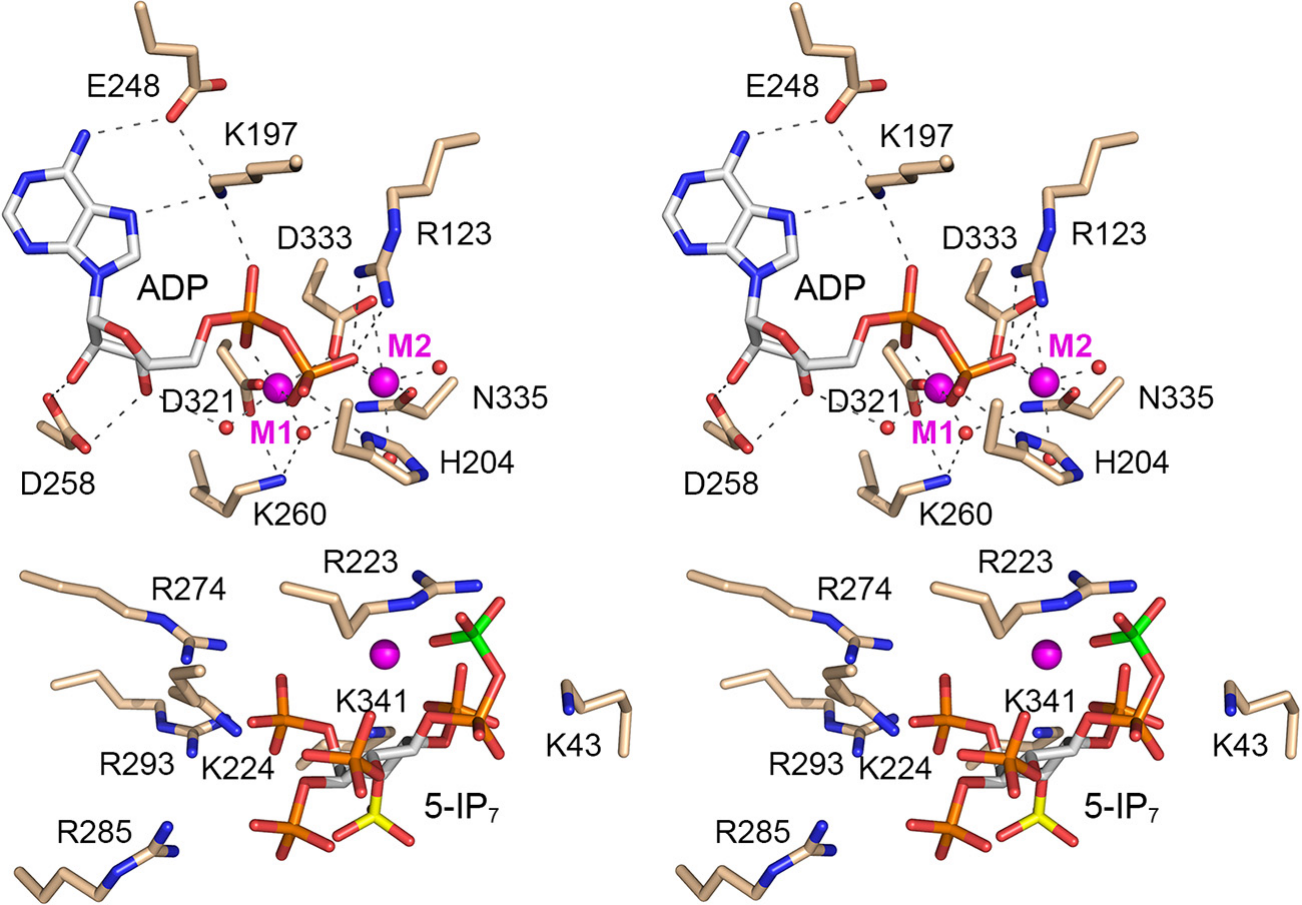
Asp1 kinase in an ATPase reaction product complex with ADP·(Mg^2+^)_2_. Stereoview of the active site of the A protomer shows selected amino acids depicted as stick models with beige carbons. ADP is rendered as a stick model with gray carbons, adjacent to two magnesium ions (M1 and M2; magenta spheres). Metal-bound waters are red spheres. Atomic contacts of ADP, Mg1, and Mg2 are indicated by dashed lines. 5-IP_7_ is a stick model with carbons in gray, the 1-phosphorus atom colored yellow, and the 5-β-phosphorus atom colored green. An IP_7_-adjacent Mg^2+^ is a magenta sphere.

We also grew crystals from a premixture of 0.1 mM Asp1-(32-364), 1 mM ATP, 5 mM Mg^2+^, and 1 mM 5-IP_7_. The aim in this case was to capture a structure of either an ATP·5-IP_7_ substrate complex or an ADP·1,5-IP_8_ product complex of the kinase reaction. A single crystal diffracting X rays to 1.9 Å resolution was used to solve the structure (Table S1). One protomer was free enzyme and the other was bound to ADP and IP_7_. The nucleotide site contained two Mg^2+^ ions adjacent to ADP. There was no observable density for a γ-phosphate. The Mg^2+^ in the M1 site was coordinated octahedrally to α- and β-phosphate oxygens, the Asp321-Oδ2 and Asp333-Oδ2 atoms, and two waters. The Mg^2+^ in the M2 site was coordinated to a β-phosphate oxygen, the Asp333-Oδ2 and -Oδ1 atoms, Asn335-Oδ, and two waters (Fig. 7). The ADP β-phosphate was engaged by His204 and Arg123; the α-phosphate was contacted by Lys197 (Fig. 7). We surmise that this structure exemplifies a product complex of the ATPase reaction catalyzed by Asp1. 5-IP_7_ was modeled as shown in Fig. 7, again in an unproductive orientation in which the 1-phosphate (depicted with a yellow phosphorus atom) was remote from the ADP nucleotide.

### Mutagenesis of the adenosine-binding site.

**FIG 8:**
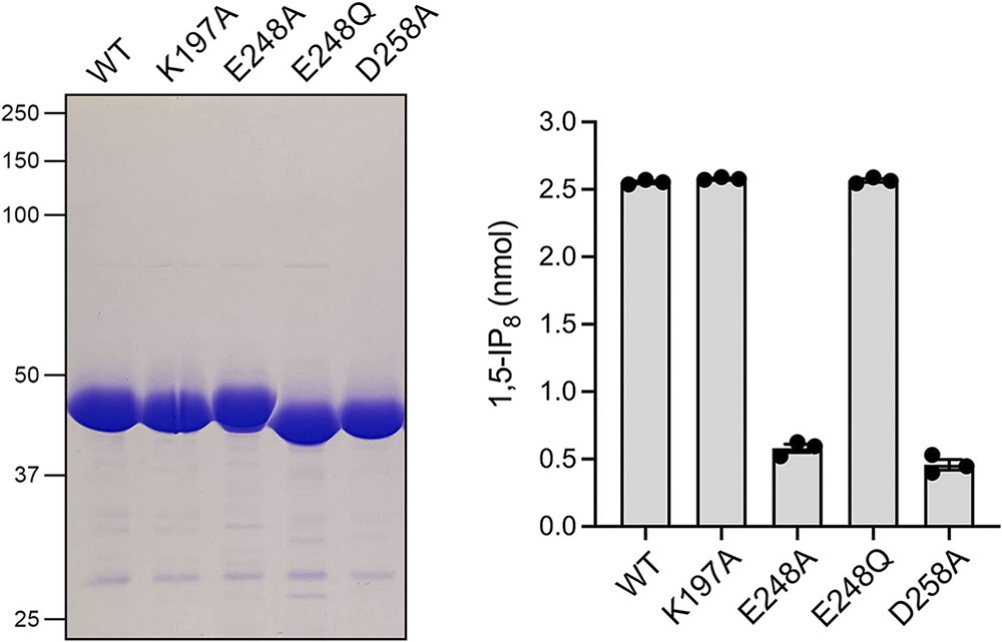
Structure-guided mutagenesis of adenosine-interacting constituents of Asp1 kinase. (Left panel) Aliquots (8 μg) of wild-type Asp1 kinase domain and the indicated mutants were analyzed by SDS-PAGE. A Coomassie blue-stained gel is shown. The positions and sizes (in kilodaltons) of marker polypeptides are indicated. (Right panel) Kinase reaction mixtures (20 μL) containing 30 mM bis-Tris (pH 6.2), 50 mM NaCl, 5 mM MgCl_2_, 0.25 mM (5 nmol) [γ-^32^P]ATP, 0.5 mM (10 nmol) 5-IP_7_, and 2.5 μM (50 pmol) of wild-type Asp1 kinase domain or the indicated mutants were incubated at 37°C for 15 min. The products were analyzed by TLC. The extents of IP_8_ formation are plotted for each enzyme. The data in the bar graph are the averages of three independent experiments ± standard errors of the means.

A detailed view of Asp1’s atomic contacts to the adenosine moiety of ADPNP is shown in Fig. S3. In addition to the hydrogen bonds from Asp258 to the ribose hydroxyls and from Glu248 and Lys197 to the adenine *N*^6^ and *N*^7^ atoms cited above, adenine-*N*^6^ engages in a hydrogen bond to the Glu249 main-chain carbonyl, and adenine *N*^1^ receives a hydrogen bond from the Met251 main chain amide (Fig. S3). The adenine nucleobase forms a hydrophobic-aromatic-hydrophobic sandwich between Val195 and Leu323, which make van der Waals contacts to the adenine-*N*^1^, -*C*^6^, -*N*^6^, and -*C*^5^ atoms (Fig. S3). Pro138 also makes van der Waals contact to adenine-*N*^6^. To query the contributions of side-chain hydrogen bonding to adenosine, we mutated Lys197, Glu248, and Asp258 individually to alanine. We also mutated Glu248 to glutamine, which would allow this side chain to serve as either hydrogen bond donor or acceptor. The mutations were introduced into the Asp1-(1-385) kinase domain expression plasmid (8), and the recombinant kinase mutants were produced in E. coli in parallel with wild-type Asp1-(1-385). SDS-PAGE analysis of the respective peak from Superdex-200 fractions is shown in Fig. 8, left panel. Equivalent amounts of wild-type and mutant proteins were assayed for kinase activity with 5-IP_7_ substrate, gauged by label transfer from [γ-^32^P]ATP to 5-IP_7_ to form ^32^P-1,5-IP_8_, which was separated from ATP by polyethyleneimine (PEI)-cellulose thin-layer chromatography (TLC) as described elsewhere (8). Whereas the K197A and E248Q proteins were as active as wild-type, the E248A and D258A mutants were 23% and 18% as active as wild-type, respectively (Fig. 8, right panel). We surmised that the hydrogen bond contacts of Glu248 to the adenine-*N*^6^ and of Asp258 to the ribose 3′-OH are important for optimal kinase activity. (We showed previously that dATP was as effective a phosphate donor as ATP [8], indicating that enzymatic contact to the 2′-OH is not critical [8]).

10.1128/mbio.03087-22.3 FIG S3 Enzymic contacts to the adenosine nucleoside. Stereoview of the phospho-donor site of the Asp1·ADPNP·Mg·IP_7_ complex highlights the atomic contacts to the adenosine moiety of ADPNP. Hydrogen bonds are denoted by black dashed lines; van der Waals contacts are magenta dashed lines. Amino acid side chains that interact with adenosine are in plain font. Amino acids that make main-chain contacts to adenosine are labeled in italics. Download FIG S3, JPG file, 0.2 MB. Copyright © 2022 Benjamin et al. 2022 Benjamin et al. https://creativecommons.org/licenses/by/4.0/ This content is distributed under the terms of the Creative Commons Attribution 4.0 International license.

### E248Q enables utilization of GTP as a phosphate donor in the kinase reaction.

**FIG 9:**
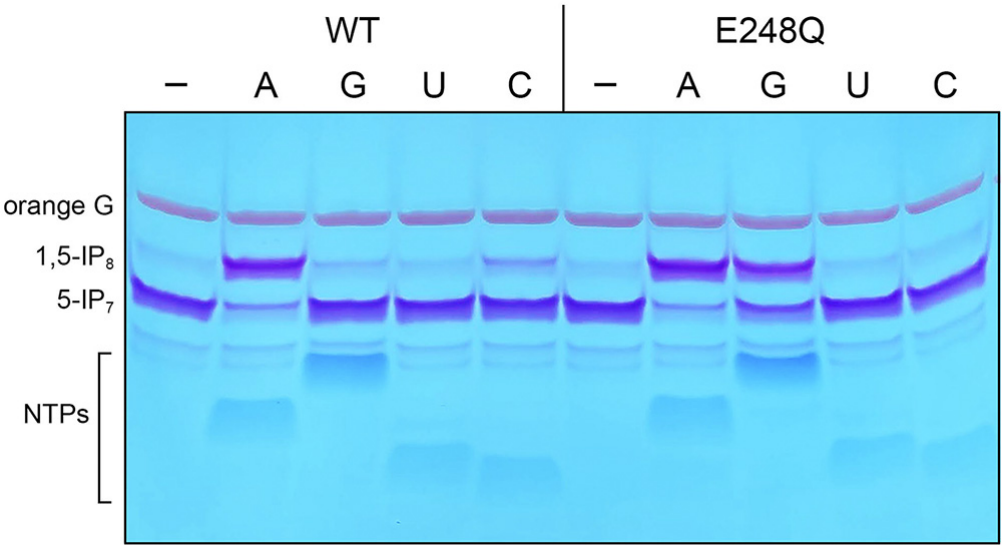
E248Q enables utilization of GTP as a phosphate donor in the kinase reaction. Reaction mixtures (20 μL) containing 30 mM bis-Tris (pH 6.2), 50 mM NaCl, 5 mM MgCl_2_, 0.5 mM 5-IP_7_, 0.5 μM (10 pmol) wild-type or E248Q Asp1 kinase, and 2 mM NTP (ATP, GTP, UTP, or CTP, as specified) were incubated at 37°C for 15 min. NTP was omitted from control reactions in lanes markd with a minus sign. The reactions were quenched by addition of 1 μL of 0.5 M EDTA. The mixtures were adjusted to 15% glycerol and 0.05% Orange G dye and analyzed by electrophoresis through a 35% polyacrylamide gel in TBE buffer (80 mM Tris-borate [pH 8.3], 1 mM EDTA) at 4°C (8 W constant power, for ~4 h). The NTPs and inositol pyrophosphates were visualized by staining the gel with toluidine blue.

Subsequent experiments focused on the contributions of Glu248 to NTP specificity, by comparing the 5-IP_7_ kinase activities of wild-type and E248Q Asp1 proteins with each of the four standard NTPs. For this experiment, we reacted 0.5 μM Asp1 kinase with 2 mM NTP, 5 mM magnesium, and 0.5 mM 5-IP_7_ for 15 min, resolved the products by PAGE, and stained the gel with toluidine blue to visualize the inositol pyrophosphates. Wild-type Asp1 kinase readily converted 5-IP_7_ to a more slowly migrating 1,5-IP_8_ product in the presence of ATP (Fig. 9). CTP was a weak phosphate donor; GTP was less effective than CTP; and UTP was unreactive (Fig. 9). The salient findings were that the E248Q mutation, which did not affect kinase activity with ATP, elicited a gain of function whereby GTP was enabled as a phosphate donor, while CTP was disabled (Fig. 9). We surmised that the amide moiety of glutamine is ambidextrous in its ability to hydrogen bond with guanine-*O*^6^ via Nε and with adenine-*N*^6^ via Oε. Assaying IP_7_ kinase at three ATP concentrations, 0.5, 1, and 2 mM, corresponding to NTP:IP_7_ ratios of 1.0, 2.0, and 4.0, indicated that wild-type and E248Q enzymes were equally adept at ATP utilization (Fig. S4). Based on the GTP concentration dependence of product formation by E248Q, we estimate that ATP is ~4-fold more effective than GTP as a kinase substrate for the mutant enzyme, i.e., substrate consumption and product formation at 2 mM GTP are similar to that at 0.5 mM ATP (Fig. S4).

10.1128/mbio.03087-22.4 FIG S4 ATP/GTP concentration dependence of E248Q kinase activity. Reaction mixtures (20 μL) containing 30 mM bis-Tris (pH 6.2), 50 mM NaCl, 5 mM MgCl_2_, 0.5 mM 5-IP_7_, 0.5 μM (10 pmol) wild-type or E248Q Asp1 kinase, and 0, 0.5, 1, or 2 mM ATP or GTP, as specified, were incubated at 37°C for 15 min. The reactions were quenched by addition of 1 μL of 0.5 M EDTA. The products were resolved by PAGE. The NTPs and inositol pyrophosphates were visualized by toluidine blue staining. Download FIG S4, JPG file, 0.2 MB. Copyright © 2022 Benjamin et al. 2022 Benjamin et al. https://creativecommons.org/licenses/by/4.0/ This content is distributed under the terms of the Creative Commons Attribution 4.0 International license.

### Wild-type Asp1 kinase can utilize *N*^6^-benzyl-ATP as a phosphate donor.

**FIG 10:**
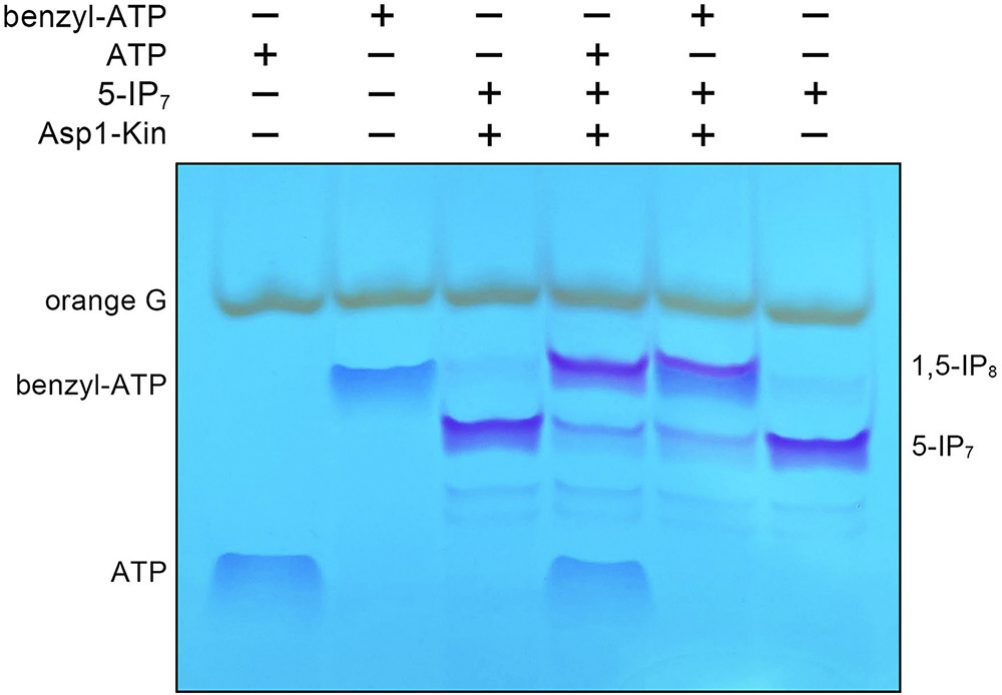
Asp1 kinase can utilize *N*^6^-benzyl-ATP as a phosphate donor. Reaction mixtures (20 μL) containing 30 mM bis-Tris (pH 6.2), 50 mM NaCl, 5 mM MgCl_2_, and 2 mM ATP or *N*^6^-benzyl-ATP (Jena Bioscience catalog number NU-1196S), 0.5 mM 5-IP_7_, and 2.5 μM (50 pmol) Asp1 kinase (indicated by +) were incubated at 37°C for 15 min. The reactions were quenched by addition of 1 μL of 0.5 M EDTA. The products were resolved by PAGE. The NTPs and inositol pyrophosphates were visualized by toluidine blue staining.

A major leap forward in understanding the physiology and pharmacology of protein kinases ensued from the engineering by Shokat and colleagues of kinase mutants that accept unnatural nucleotides as phosphate donor substrates. For example, Liu et al. (28) described a single I338G mutant of v-Src kinase that preferentially utilized *N*^6^-benzyl-ATP over ATP, in contrast to wild-type v-Src, which was unable to utilize *N*^6^-benzyl-ATP. As an opening foray into this area for Asp1 kinase, we queried its ability to accept *N*^6^-benzyl-ATP as a phospho-donor. Our initial expectation that *N*^6^-benzyl-ATP would not work, given the apparent lack of space in the vicinity of adenine-*N*^6^ in the present Asp1 crystal structures that could accommodate a bulky *N*^6^-benzyl moiety (Fig. S3). ATP and *N*^6^-benzyl-ATP displayed distinctive electrophoretic mobilities during the PAGE analysis employed for our kinase assays (Fig. 10). To our surprise, we found that Asp1 kinase was adept at converting 5-IP_7_ to 1,5-IP_8_ in the presence of *N*^6^-benzyl-ATP (Fig. 10).

### Structure of Asp1 in complex with 1,5-IP_8_.

**FIG 11:**
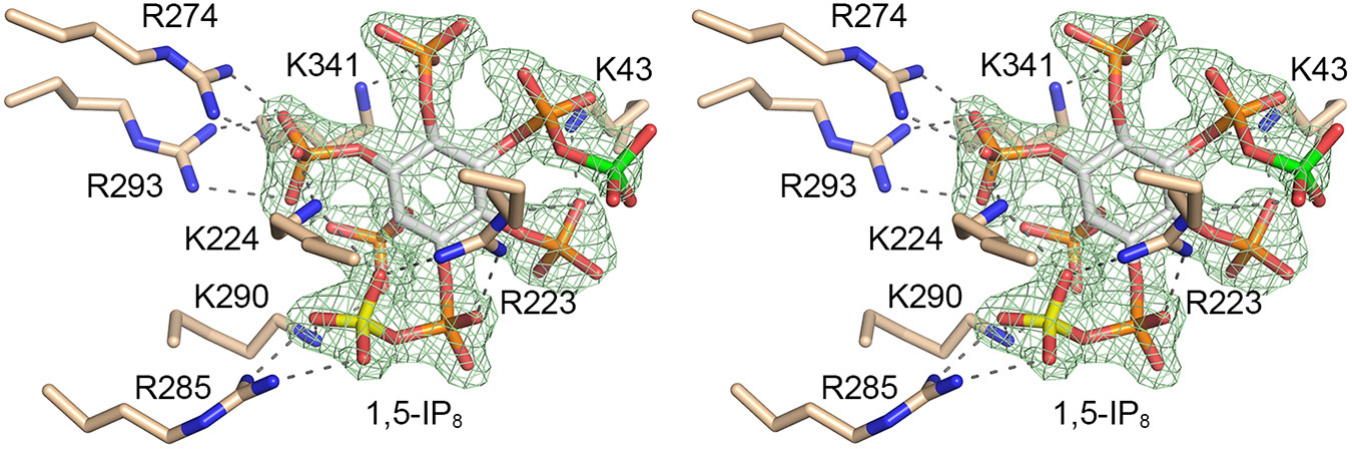
Asp1 kinase in complex with 1,5-IP_8_. Stereoview of the IP_8_ site in the A protomer with amino acids depicted as stick models with beige carbons. 1,5-IP_8_ is rendered as a stick model with carbons in gray, the 1-β-phosphorus atom colored yellow, and the 5-β-phosphorus atom colored green. An omit density maps overlying 1,5-IP_8_ is contoured at 1σ and depicted as a green mesh. Atomic contacts to 1,5-IP_8_ are indicated by dashed lines.

A crystal of Asp1 kinase grown from a premixture with *N*^6^-benzyl-ATP, Mg^2+^, and 5-IP_7_ diffracted to 1.6 Å resolution and contained two protomers in the asymmetric unit, one ligand-free and one bound to the reaction product, 1,5-IP_8_. The fit of IP_8_ into the omit electron density map is shown in Fig. 11. To our surprise, the adenine nucleotide-binding site was unoccupied. Because IP_8_ blocks access to, or exit from, the ATP-binding pocket, we presume that the kinase in solution catalyzed phospho-transfer from *N*^6^-benzyl-ATP to 5-IP_7_ and both products dissociated, after which the IP_8_ reassociated with the inositol polyphosphate site, to yield the “closed” kinase protomer that crystallized. Moreover, in the absence of nucleotide occupancy, the peptide segment of the adenosine-binding pocket of the A protomer from aa 252 to 257 was disordered. The 8 phosphates of the 1,5-IP_8_ molecule are encaged by atomic contacts to basic side chains Lys43, Arg223, Lys224, Arg274, Arg285, Lys290, Arg293, and Lys341. Superposition of the IP_8_-bound A protomer on an ADPNP-bound A protomer showed that 1,5-IP_8_ is oriented such that the 1-pyrophosphate is remote and pointing away from the nucleotide phosphates (the 1-β-phosphate of IP_8_ is 12 Å from the ADPNP β-phosphate), signifying that the 1,5-IP_8_ ligand is not bound in a state mimetic of the immediate product of the 5-IP_7_ kinase reaction.

### An on-pathway complex of Asp1 with ATP and 5-IP_7_.

**FIG 12:**
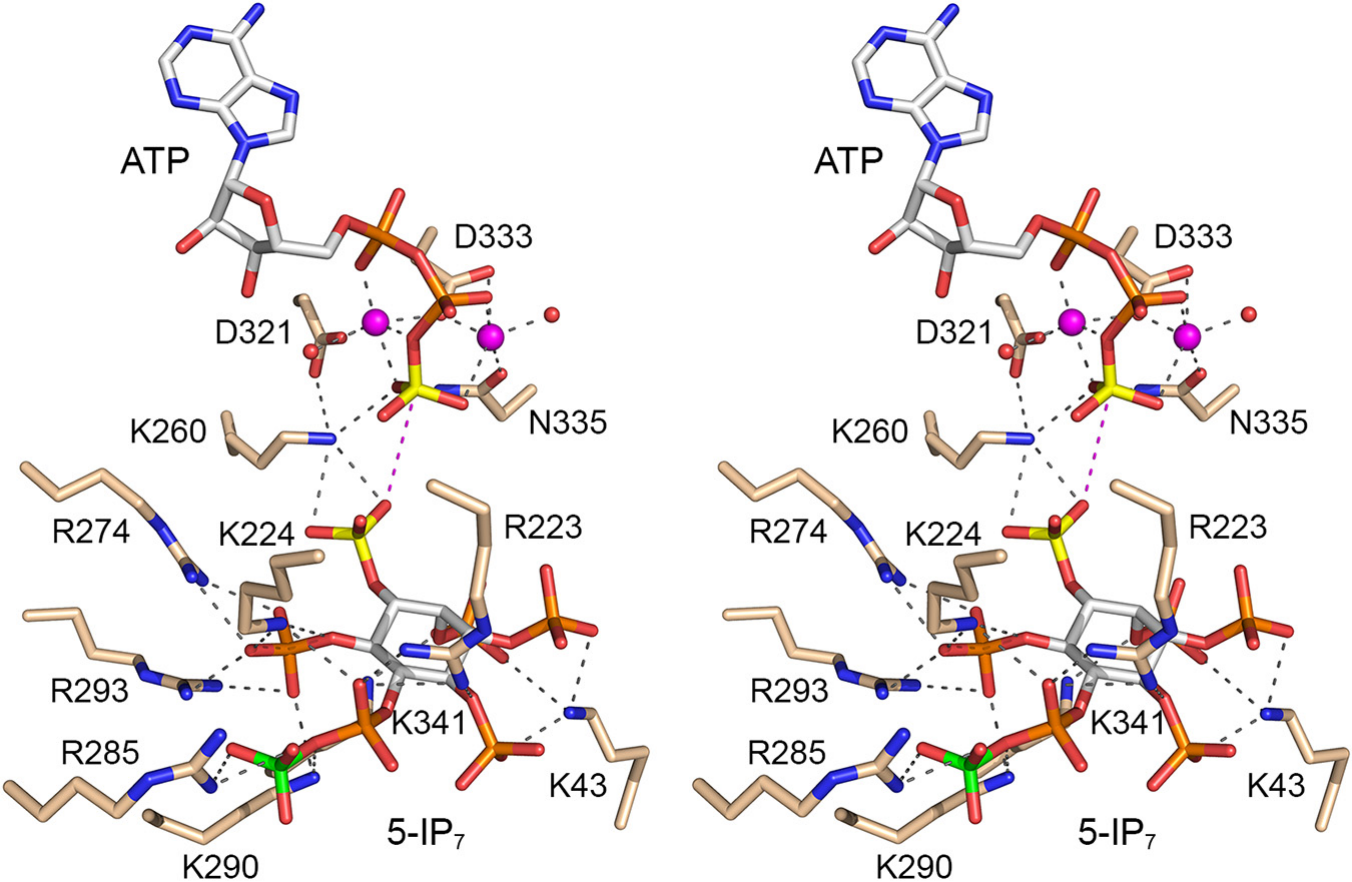
Michaelis-like complex of Asp1 with ATP and 5-IP_7_. Stereoview of the active site of the A protomer with selected amino acids depicted as stick models with beige carbons. ATP is rendered as a stick model with gray carbons and a yellow γ-phosphorus atom, adjacent to two magnesium ions (magenta spheres). 5-IP_7_ is a stick model with carbons in gray, the 1-phosphorus atom colored yellow, and the 5-β-phosphorus atom colored green. Atomic contacts of ATP·Mg^2+^ and 5-IP_7_ are indicated by dashed lines. The distance (4.4 Å) from the ATP γ-phosphorus to the closest 1-phosphate oxygen of 5-IP_7_ is denoted by a magenta dashed line.

The open and closed conformations of the kinase protomers are stabilized by atomic contacts between the A and B protomers within the asymmetric unit and by packing against, and atomic contacts to, kinase protomers in adjacent asymmetric units of the crystal lattice. Of note, within the asymmetric unit, the Arg50 and Lys47 side chains of the B protomer are adjacent to the 5-IP_7_ and 1,5-IP_8_ ligands bound to the A protomer, whereby Arg50 and Lys47 make interprotomer atomic contacts to inositol phosphate groups, potentially constraining the full penetration of the inositol polyphosphate ligands into the active site and adoption of a proper on-pathway orientation. To overcome this potential constraint, we produced and purified a K47A-R50A double mutant of the Asp1-(31-364) kinase domain, verified that it was catalytically active in converting 5-IP_7_ to 1,5-IP_8_, and conducted crystallization trials with this version of the kinase domain, with various ligands and precipitant conditions. A crystal grown by mixing a solution of 0.15 mM Asp1-(K47A-R50A), 10 mM magnesium, 1 mM ATP, and 1 mM 5-IP_7_ with precipitant containing 0.1 M bis-Tris (pH 7.8), 0.1 M NaF, and 20% PEG 3350 diffracted to 2.0 Å. The asymmetric unit included a ligand-bound A protomer and a ligand-free B protomer. The salient point was that the A protomer contained ATP·(Mg^2+^)_2_ in the nucleotide pocket and 5-IP_7_ in the substrate site, in a state mimetic of a Michaelis complex for the kinase reaction. To wit, a 1-phosphate oxygen of 5-IP_7_ was positioned 4.4 Å from the γ-phosphorus at an O-Pγ-O angle of 162° to the ADP leaving group (Fig. 12). Thus, the proximity and geometry of the ATP and 5-IP_7_ substrates were improved (i.e., closer to on-pathway) compared to the same parameters in the ADPNP/IP_6_ substrate complex (distance of 4.8 Å; angle of 143°). Superposition of the ATP/5-IP_7_ and ADPNP/IP_6_ structures showed that the orientation of the inositol polyphosphate ring and the Lys/Arg contacts to the inositol phosphates were similar, except that, in the 5-IP_7_ complex, Arg285 engaged the 5-β-phosphate while Arg223 contacted the 5-α-phosphate and the 4-phosphate. The position of the inositol ring was shifted by ~1 Å in the ATP/5-IP_7_ complex compared to that in the ADPNP/IP_6_ structure.

## DISCUSSION

The results herein fortify our understanding of the structure, mechanism, and substrate specificity of the fission yeast Asp1 kinase, and they extend and complement previous structural and functional studies by the Shears lab of the orthologous human kinase PPIP5K2.

We report an ensemble of structures of a catalytically active N-terminally truncated kinase domain, Asp1-(31-364), in complex with nucleotides ADPNP, ADP, and ATP, divalent cations magnesium and manganese, and inositol polyphosphates IP_6_, 5-IP_7_, and 1,5-IP_8_. All crystals contained two kinase protomers in the asymmetric unit that differed in their conformations and active site occupancies. The “open” conformation seen for the ligand-free kinase was amenable to ingress of the nucleotide phospho-donor. Yet, the nucleotide site was either empty or contained very weak electron density for adenine (interpreted as very low partial occupancy) in the dozens of crystals for which we collected diffraction data, suggesting that ADPNP or ATP, if initially bound, can readily leach out of the active site of this protomer. A plausible contributing factor to the absence of nucleotide in the open protomer was suggested by the superimposition of the ADPNP-bound and nucleotide-free A and B protomers (see Fig. S5 in the supplemental material [with A and B protomers colored gray and cyan, respectively]), which indicated that the peptide segment between Glu248 and Asp258, which comprises part of the adenosine-binding pocket, moved and adopted a different conformation in the B protomer, entailing a 6.8-Å displacement at Asp254-Cα that was apparently imposed by lattice contacts, including a salt bridge from Asp254 to Arg161 of the A promoter in a neighboring asymmetric unit. An upshot of this difference was that the Met251-N and Glu249-O main chain atoms now clashed with the adenine ring.

10.1128/mbio.03087-22.5 FIG S5 Overlay of the adenine-binding sites of the A and B protomers. Stereoviews of the superimposed adenine binding sites of the ligand-free B protomer (colored cyan) and the ADPNP-bound A protomer (colored gray) that comprise the asymmetric unit of the ADPNP·IP6 crystal are shown. The conformation of the loop from aa 249 to 257 differs in the nucleotide-bound versus free states. Amino acids that contact adenosine or undergo displacement in A versus B protomers are depicted as stick models. Hydrogen bond contacts to adenine in the A protomer are denoted by black dashed lines. The Cα-to-Cα displacements of Asn252 (3.6 Å), Val253 (5.2 Å), Glu254 (6.8 Å), and Asn255 (3.5 Å) are denoted by green dashed lines. Download FIG S5, JPG file, 0.2 MB. Copyright © 2022 Benjamin et al. 2022 Benjamin et al. https://creativecommons.org/licenses/by/4.0/ This content is distributed under the terms of the Creative Commons Attribution 4.0 International license.

The “closed” conformation adopted by the ligand-bound kinase A protomer entailed a disordered-to-ordered transition and a conformational rearrangement or movement of two peptide segments that formed a binding site for the IP_6_/IP_7_ phospho-acceptor. The fact that the phospho-acceptor sits near the protein surface on top of the deeply buried nucleotide ensures that the phospho-donor site remains occupied in the crystal and virtually mandates an ordered bi-bi reaction mechanism in which ATP binds prior to IP_6_/5-IP_7_ substrate and 1-IP_7_/1,5-IP_8_ product dissociates prior to ADP. Our Asp1 structures varied as to whether they contained one or two metal ions in complex with the nucleotide. Taking into account that PPIP5K2 structures consistently have two metals adjacent to the nucleotide (16) and that the enzymatic contacts between Asp1 kinase and the M1 and M2 metals are conserved in PPIP5K2, we regarded the two-metal state as on-pathway for the Asp1 kinase and phosphohydrolase reactions.

Whereas the crystals captured two static snapshots of alternative Asp1 conformations, we envision that in solution the kinase can sample a continuum between open and closed states, with a “fully closed” conformation being that of the Michaelis complex. Several of the inositol polyphosphate-bound structures reported here do not correspond to a Michaelis complex mimetic, insofar as the 1-phosphate moiety is too far from the ADPNP γ-phosphate to initiate a nucleophilic attack and the inositol polyphosphate ring is unproductively oriented. We regard such structures as being incompletely closed, such that more than one substrate orientation can be accommodated by the ensemble of cationic residues that surround the anionic inositol polyphosphate ligands. Inspection of the Asp1 crystal lattice indicated that the open and incompletely closed conformations of the kinase protomers were stabilized by atomic contacts between the A and B protomers within the asymmetric unit and by packing against, and atomic contacts to, kinase promoters in adjacent asymmetric units. By contrast, crystals of PPIP5K2, which were grown initially with ADPNP or ATP and then soaked in a solution containing magnesium and 5-IP_7_ or IP_6_, had one protomer in the asymmetric unit and yielded structures of the human kinase in a fully closed conformation with ADPNP·IP_7_/IP_6_ (a Michaelis-like complex) and with ADP·MgF_3_·5-IP_7_ (a transition-state analog) (16).

By deploying a mutated version of the Asp1 kinase that eliminated contacts of B protomer Lys47 and Arg50 with the inositol polyphosphate ligand in the A protomer, we obtained a crystal in which the nucleotide and substrate sites were occupied by ATP·(Mg^2+^)_2_ and 5-IP_7_ in an on-pathway state that approximated a Michaelis complex of the kinase reaction. Here, the 5-IP_7_ substrate was engaged by 9 cationic amino acids (Lys43, Arg223, Lys224, Lys260, Arg274, Arg285, Lys290, Arg293, and Lys341) (Fig. 12).

The unproductively oriented 5-IP_7_ and 1,5-IP_8_ ligands in our Asp1 crystals can be viewed as analogous to the inositol polyphosphate ligands occupying a substrate capture site in PPIP5K2, which is too far from ATP to allow catalysis but which overlaps the productive binding site for the phospho-acceptor (17). PPIP5K2 amino acid side chains Lys53, Lys103, Glu192, and His194 specifically contact the ligand in the capture site; the latter two of these are conserved in Asp1 (as Glu202 and His204).

Asp1 kinase utilizes ATP and dATP as the phosphate donor and is either inactive or feebly active with GTP, CTP, and UTP (8) (Fig. 9). The adenine requirement is consistent with the nucleobase-specific enzymatic contacts evident in the Asp1 crystal structures. By replacing the three side chains that make hydrogen bonds to the adenosine nucleoside with alanine, we established that Asp258 and Glu248 each enhanced kinase activity by 4- to 5-fold, via their contacts with the ribose hydroxyls and adenine-*N*^6^, respectively, whereas Lys197, which made bifurcated contacts to adenine-*N*^7^ and the nucleotide α-phosphate, was apparently noncontributory under the kinase assay conditions employed. The key finding here was that replacing Glu248 with glutamine conferred a neomorphic phenotype whereby the E248Q enzyme was able to utilize GTP as a phospho-donor, while suffering no loss of activity with ATP, presumably because the glutamine amide nitrogen could donate a favorable hydrogen bond to guanine-*O*^6^. The resulting implication that the nucleoside binding side of Asp1 is plastic received confirmation from the initially surprising observation that the kinase was active with *N*^6^-benzyl-ATP in lieu of ATP.

## MATERIALS AND METHODS

### Recombinant Asp1 kinase domain.

Plasmid pET28b-His_10_Smt3-Asp1-(31-364), carrying an N-terminally truncated version of the Asp1 kinase domain fused to an N-terminal His_10_Smt3 tag, was transformed into Escherichia coli BL21(DE3). A culture (3.2 liters) amplified from a single transformant was grown at 37°C in Terrific broth (Invitrogen) containing 50 μg/mL kanamycin until the *A*_600_ reached 0.8, and then was adjusted to 2% (vol/vol) ethanol and placed on ice for 30 min. Asp1 kinase expression was induced by adding isopropyl-β-d-1-thiogalactopyranoside to 0.25 mM and incubating the cultures overnight at 17°C with constant shaking. Cells were harvested by centrifugation and resuspended in buffer A (50 mM Tris-HCl [pH 8.0], 500 mM NaCl, 10% glycerol) containing 10 mM imidazole and one cOmplet protease inhibitor cocktail tablet (Roche) at a volume of 25 mL per liter of culture. All subsequent purification procedures were performed at 4°C. Cell lysis was achieved by adding lysozyme to 0.5 mg/mL and incubating for 1 h, followed by sonication to reduce viscosity. The lysate was centrifuged at 38,000 × *g* for 45 min, and the supernatant was mixed with 5 mL of Ni-nitrilotriacetic acid–agarose resin (Qiagen) that had been equilibrated in buffer A with 10 mM imidazole. After 1 h of mixing on a nutator, the resin was recovered by centrifugation and washed twice with 50 mL of buffer A containing 20 mM imidazole. The washed resin was poured into a column, and the bound protein was eluted with 250 mM imidazole in buffer A. The elution of His_10_Smt3-Asp1-(31-364) protein was monitored by SDS-PAGE. The His_10_Smt3 tag was cleaved by treatment with Ulp1 protease (100 μg Ulp1 per liter of bacterial culture) during overnight dialysis against buffer A with 20 mM imidazole. Asp1-(31-364) was separated from the His_10_Smt3 tag by a second round of Ni-affinity chromatography, during which Asp1-(31-364) was recovered in the flowthrough fraction. Tag-free Asp1-(31-364) was concentrated to a volume of 5 mL by centrifugal ultrafiltration and then applied to a Hiload Superdex 200 pg 16/600 column (Cytiva Life Sciences) equilibrated in buffer B (30 mM HEPES [pH 6.8], 100 mM NaCl, 1 mM dithiothreitol). The peak Superdex fractions of Asp1-(31-364) were concentrated by centrifugal ultrafiltration and stored at −80°C. Protein concentrations were determined by using the Bio-Rad dye reagent with bovine serum albumin as the standard. Recombinant Asp1-(31-364) catalyzed the ATP·Mg^2+^-dependent conversion of 5-IP_7_ to 1,5-IP_8_, which was assayed as described previously (8).

Asp1-(1-385) and mutant kinases Asp1-(31-364)-K47A-R50A, Asp1-(1-385)-D258A, Asp1-(1-385)-K197A, Asp1-(1-385)-E248A, and Asp1-(1-385)-E248Q were produced in E. coli and purified via two rounds of Ni-affinity chromatography and then Superdex 200 gel filtration as described above.

### Crystallization.

**(i) Asp1·ADPNP·Mg^2+^·IP_6_ complex.** A solution containing 4 mg/mL Asp1 kinase, 5 mM MgCl_2_, 1 mM ADPNP, and 1 mM IP_6_ (phytic acid; Sigma catalog number P-8810-10G) was preincubated on ice for 30 min. Aliquots (3 μL) were mixed with 3 μL of precipitant solution containing 0.1 M bis-Tris (pH 5.5), 0.1 M NH_4_OAc, and 17% (wt/vol) PEG 10,000. Crystals were grown by sitting -drop vapor diffusion at room temperature. Crystals grew over 1 week and were harvested within 1 week of observation. Crystals were cryoprotected with precipitant solution containing 25% ethylene glycol for 1 min and then flash-frozen in liquid nitrogen.

**(ii) Asp1·ADPNP·Mn^2+^·IP_6_ complex.** A solution containing 4 mg/mL Asp1 kinase, 5 mM MnCl_2_, 1 mM ADPNP, and 1 mM IP_6_ was preincubated on ice for 30 min. Aliquots (3 μL) were mixed with 3 μL of precipitant solution containing 0.1 M bis-Tris (pH 5.9), 0.025 M NH_4_OAc, and 17% PEG 10,000. Crystals were grown by sitting-drop vapor diffusion at room temperature. A single crystal grew over 1 week, which was then manually crushed and the well was resealed. Additional crystals seeded from the crushed crystal shards grew over the next week and were harvested. Crystals were cryoprotected with precipitant solution containing 25% ethylene glycol and 5 mM MnCl_2_ for 1 min and then flash-frozen in liquid nitrogen.

**(iii) Asp1·ADPNP·Mg^2+^·5-IP_7_ complex.** A solution containing 4 mg/mL Asp1 kinase, 5 mM MgCl_2_, 1 mM ADPNP, and 1 mM 5-IP_7_ (synthesized as described elsewhere [29]) was preincubated on ice for 30 min. Aliquots (2 μL) were then mixed with 2 μL of precipitant solution containing 0.1 M bis-Tris propane (pH 8.4), 0.15 M NaF, and 20% PEG 3350. Crystals were grown by sitting-drop vapor diffusion at room temperature. Crystals grew over 1 week and were harvested within 1 week of observation. Crystals were cryoprotected with precipitant solution containing 25% glycerol, 5 mM MgCl_2_, 1 mM ADPNP, and 1 mM 5-IP_7_ for 1 min and then flash-frozen in liquid nitrogen.

**(iv) Asp1·ADP·Mg^2+^·5-IP_7_ complex.** A solution containing 4 mg/mL Asp1 kinase, 5 mM MgCl_2_, 1 mM ATP, and 1 mM 5-IP_7_ was preincubated on ice for 30 min. Aliquots (2 μL) were then mixed with 2 μL of precipitant solution containing 0.1 M bis-Tris propane (pH 8.1), 0.15 M NaF, and 20% PEG 3350. Crystals were grown by sitting-drop vapor diffusion at room temperature. Crystals grew over 1 week and were harvested within 1 week of observation. Crystals were cryoprotected with precipitant solution containing 25% glycerol, 5 mM MgCl_2_, 1 mM ATP, and 1 mM 5-IP_7_ for 1 min and then flash-frozen in liquid nitrogen.

**(v) Asp1·1,5-IP_8_ complex**. A solution containing 4 mg/mL Asp1 kinase, 10 mM MgCl_2_, 1 mM *N*^6^-benzyl ATP (Jena Biosciences), and 1 mM 5-IP_7_ was preincubated at room temperature for 30 min. Aliquots (2 μL) were then mixed with 2 μL of precipitant solution containing 0.1 M bis-Tris propane (pH 6.9), 0.3 M NaF, and 20% PEG 3350. Crystals were grown by sitting-drop vapor diffusion at room temperature. Crystals grew over 1 week and were harvested within 1 week of observation. Crystals were cryoprotected with precipitant solution containing 25% ethylene glycol, 50 mM bis-Tris propane (pH 6.9), 0.1 M NaF, 10 mM MgCl_2_, and 20% PEG 3350 for 1 min and then flash-frozen in liquid nitrogen.

**(vi) Asp1-(K47A-R50A)·ATP·Mg^2+^·5-IP_7_ complex.** A solution containing 6 mg/mL Asp1-(K47A-R50A) kinase, 10 mM MgCl_2_, 1 mM ATP, and 1 mM 5-IP_7_ was preincubated at room temperature for 30 min. Aliquots (2 μL) were then mixed with 2 μL of precipitant solution containing 50 mM bis-Tris propane (pH 7.8), 0.1 M NaF, and 20% PEG 3350. Crystals were grown by sitting-drop vapor diffusion at room temperature. Crystals grew over 1 week and were harvested within 1 week of observation. Crystals were cryoprotected with precipitant solution containing 25% ethylene glycol, 50 mM bis-Tris propane (pH 6.6), 0.1 M NaF, 10 mM MgCl_2_, and 20% PEG 3350 for 1 min and then flash-frozen in liquid nitrogen.

### Diffraction data collection and structure determination.

X-ray diffraction data were collected from single crystals at APS beamline 24ID-E. The data were integrated with HKL2000 (30). Phases were obtained in Phenix (31) by molecular replacement using a Phyre2 model (32) templated on the PPIP5K2 kinase domain (PDB ID 4NZM) as the search probe. Iterative model building into electron density was performed with O (33). Refinement was accomplished with Phenix. Data collection and refinement statistics are presented in Table S1 in the supplemental material.

### TLC assay of Asp1 kinase activity.

Reaction mixtures containing 30 mM bis-Tris (pH 6.2), 50 mM NaCl, 5 mM MgCl_2_, [γ-^32^P]ATP, 5-IP_7_, and Asp1-(1-385) at concentrations specified in the figure legends were incubated at 37°C. Reactions were initiated by addition of Asp1 and quenched at the times specified by adjustment to 25 mM EDTA. Aliquots (2 μL) were applied to a PEI-cellulose TLC plate (Millipore-Sigma), and the products were resolved by ascending TLC with 1.7 M ammonium sulfate as the mobile phase. The radiolabeled ATP substrate and P_i_ and inositol pyrophosphate products were visualized and quantified by scanning the TLC plate with a Typhoon FLA7000 imager and ImageQuant-TL software.

### PAGE assay of Asp1 kinase activity.

Reaction mixtures (20 μL) containing 30 mM bis-Tris (pH 6.2), 50 mM NaCl, 5 mM MgCl_2_, NTP, and 5-IP_7_, as specified in the figure legends, were incubated at 37°C. Reactions were terminated at the times specified by adjustment to 25 mM EDTA. The samples were mixed with an equal volume of 2× Orange G loading buffer (10 mM Tris-HCl [pH 7.0], 1 mM EDTA, 30% glycerol, 0.1% Orange G dye) and then analyzed by electrophoresis (at 4°C at 8 W constant power) through a 20-cm 36% polyacrylamide gel containing 80 mM Tris-borate (pH 8.3), 1 mM EDTA until the Orange G dye reached 2/3 of the length of the gel. The gel was briefly washed with water and then stained with a solution of 0.1% toluidine blue (Sigma), 20% methanol, 2% glycerol, followed by destaining in 20% methanol.

### Data availability.

The coordinates for the refined models of Asp1 kinase have been deposited in the RCSB Protein Databank under PDB ID codes 8E1V, 8E1T, 8E1S, 8E1H, 8E1J, and 8E1I. All other data are contained within the manuscript and supporting information.

## References

[1] Sanchez AM, Garg A, Shuman S, Schwer B. 2019. Inositol pyrophosphates impact phosphate homeostasis via modulation of RNA 3’ processing and transcription termination. Nucleic Acids Res 47:8452–8469. doi:10.1093/nar/gkz567. 31276588 PMC6895273

[2] Carter-O'Connell I, Peel MT, Wykoff DD, O'Shea EK. 2012. Genome-wide characterization of the phosphate starvation response in *Schizosaccharomyces pombe*. BMC Genomics 13:697. doi:10.1186/1471-2164-13-697. 23231582 PMC3556104

[3] Shuman S. 2020. Transcriptional interference at tandem lncRNA and protein-coding genes: an emerging theme in regulation of cellular nutrient homeostasis. Nucleic Acids Res 48:8243–8254. doi:10.1093/nar/gkaa630. 32720681 PMC7470944

[4] Shears SB, Wang H. 2019. Inositol phosphate kinases: expanding the biological significance of the universal core of the protein kinase fold. Adv Biol Regul 71:118–127. doi:10.1016/j.jbior.2018.10.006. 30392847 PMC9364425

[5] Randall TA, Gu C, Li X, Wang H, Shears SB. 2020. A two-way switch for inositol pyrophosphate signaling: evolutionary history and biological significance of a unique, bifunctional kinase/phosphatase. Adv Biol Regul 75:100674. doi:10.1016/j.jbior.2019.100674. 31776069 PMC9383039

[6] Pascual-Ortiz M, Saiardi A, Walla E, Jakopec V, Künzel NA, Span I, Vangala A, Fleig U. 2018. Asp1 bifunctional activity modulates spindle function via controlling cellular inositol pyrophosphate levels in *Schizosaccharomyces pombe*. Mol Cell Biol 38:e00047-18. doi:10.1128/MCB.00047-18. 29440310 PMC5902593

[7] Dollins DE, Bai W, Fridy PC, Otto JC, Neubauer JL, Gattis SG, Mehta KP, York JD. 2020. Vip1 is a kinase and pyrophosphatase switch that regulates inositol diphosphate signaling. Proc Natl Acad Sci USA 117:9356–9364. doi:10.1073/pnas.1908875117. 32303658 PMC7196807

[8] Benjamin B, Garg A, Jork N, Jessen HJ, Schwer B, Shuman S. 2022. Activities and structure-function analysis of fission yeast inositol pyrophosphate (IPP) kinase-pyrophosphatase Asp1 and its impact of regulation of *pho1* gene expression. mBio 13:e01034-22. doi:10.1128/mbio.01034-22. 35536002 PMC9239264

[9] Osada S, Kageyama K, Ohnishi Y, Nishikawa J, Nishihara T, Imagawa M. 2012. Inositol phosphate kinase Vip1p interacts with histone chaperone Asp1p in *Saccharomyces cerevisiae*. Mol Biol Rep 39:4989–4996. doi:10.1007/s11033-011-1295-z. 22160571

[10] Lev S, Li C, Desmarini D, Saiardi A, Fewings NL, Schibeci SD, Sharma R, Sorrell TC, Djordjevic JT. 2015. Fungal inositol pyrophosphate IP_7_ is crucial for metabolic adaptation to the host environment and pathogenicity. mBio 6:e00531-15. doi:10.1128/mBio.00531-15. 26037119 PMC4453010

[11] Wilson MS, Jessen HJ, Saiardi A. 2019. The inositol hexakisphosphate kinases IP6K1 and -2 regulate human cellular phosphate homeostasis, including XPR1-mediated phosphate export. J Biol Chem 294:11597–11608. doi:10.1074/jbc.RA119.007848. 31186349 PMC6663863

[12] Zhu J, Lau K, Puschmann R, Harmel RK, Zhang Y, Pries V, Gaugler P, Broger L, Dutta AK, Jessen HJ, Schaaf G, Fernie AR, Hothorn LA, Fiedler D, Hothorn M. 2019. Two bifunctional inositol pyrophosphate kinases/phosphatases control plant phosphate homeostasis. Elife 8:e43582. doi:10.7554/eLife.43582. 31436531 PMC6731061

[13] Fridy PC, Otto JC, Dollins DE, York JD. 2007. Cloning and characterization of two human VIP1-like inositol hexakisphosphate and diphosphoinositol pentakisphosphate kinases. J Biol Chem 282:30754–30762. doi:10.1074/jbc.M704656200. 17690096

[14] Mulugu S, Bai W, Fridy PC, Bastidas RJ, Otto JC, Dollins DE, Haystead TA, Ribeiro AA, York JD. 2007. A conserved family of enzymes that phosphorylate inositol hexakisphosphate. Science 316:106–109. doi:10.1126/science.1139099. 17412958

[15] Weaver JD, Wang H, Shears SB. 2013. The kinetic properties of a human PPIP5K reveal that its kinase activities are protected against the consequences of a deteriorating cellular bioenergetic environment. Biosci Rep 33:e00022. doi:10.1042/BSR20120115. 23240582 PMC3564036

[16] Wang H, Falck JR, Tanaka Hall TM, Shears SB. 2011. Structural basis for an inositol pyrophosphate kinase surmounting phosphate crowding. Nat Chem Biol 8:111–116. doi:10.1038/nchembio.733. 22119861 PMC3923263

[17] Wang H, Godage HY, Riley AM, Weaver JD, Shears SB, Potter BV. 2014. Synthetic inositol phosphate analogs reveal that PPIP5K2 has a surface-mounted substrate capture site that is a target for drug discovery. Chem Biol 21:689–699. doi:10.1016/j.chembiol.2014.03.009. 24768307 PMC4085797

[18] Riley AM, Wang H, Shears SB, Potter BV. 2015. Synthetic tools for studying the chemical biology of InsP_8_. Chem Commun (Camb) 51:12605–12608. doi:10.1039/c5cc05017k. 26153667 PMC4643724

[19] Holm L, Kaariainen S, Rosenstrom P, Schenkel A. 2008. Searching protein structure databases with DaliLite v.3. Bioinformatics 24:2780–2781. doi:10.1093/bioinformatics/btn507. 18818215 PMC2639270

[20] Fawaz MV, Topper ME, Firestine SM. 2011. The ATP-grasp enzymes. Bioorg Chem 39:185–191. doi:10.1016/j.bioorg.2011.08.004. 21920581 PMC3243065

[21] Arimura Y, Kono T, Kino K, Kurumizaka H. 2018. Structural polymorphism of the *Escherichia coli* poly-α-L-glutamate synthetase RimK. Acta Crystallogr F Struct Biol Commun 74:385–390. doi:10.1107/S2053230X18007689. 29969101 PMC6038451

[22] Chamberlain PP, Qian X, Stiles AR, Cho J, Jones DH, Lesley SA, Grabau EA, Shears SB, Spraggon G. 2007. Integration of inositol phosphate signaling pathways via human ITPK1. J Biol Chem 282:28117–28125. doi:10.1074/jbc.M703121200. 17616525 PMC2244811

[23] Miller GJ, Wilson MP, Majerus PW, Hurley JH. 2005. Specificity determinants in inositol polyphosphate synthesis: crystal structure of inositol 1,3,4-trisphosphate 5/6-kinase. Mol Cell 18:201–212. doi:10.1016/j.molcel.2005.03.016. 15837423

[24] Matoba Y, Uda N, Kudo M, Sugiyama M. 2020. Cyclization mechanism catalyzed by an ATP-grasp enzyme essential for d-cycloserine biosynthesis. FEBS J 287:2763–2778. doi:10.1111/febs.15163. 31793174

[25] Brautigam CA, Chelliah Y, Deisenhofer J. 2004. Tetramerization and ATP binding by a protein comprising the A, B, and C domains of rat synapsin I. J Biol Chem 279:11948–11956. doi:10.1074/jbc.M312015200. 14688264

[26] Song I, Kim Y, Yu J, Go SY, Lee HG, Song WJ, Kim S. 2021. Molecular mechanism underlying substrate recognition of the peptide macrocyclase PsnB. Nat Chem Biol 17:1123–1131. doi:10.1038/s41589-021-00855-x. 34475564

[27] Diaz-Saez L, Torrie LS, McElroy SP, Gray D, Hunter WN. 2019. *Burkholderia pseudomallei* d-alanine-d-alanine ligase; detailed characterisation and assessment of a potential antibiotic drug target. FEBS J 286:4509–4524. doi:10.1111/febs.14976. 31260169 PMC6899670

[28] Liu Y, Shah K, Yang F, Witucki L, Shokat KM. 1998. Engineering Src family protein kinases with unnatural nucleotide specificity. Chem Biol 5:91–101. doi:10.1016/s1074-5521(98)90143-0. 9495830

[29] Pavlovic I, Thakor DT, Vargas JR, McKinlay CJ, Hauke S, Anstaett P, Camuna RC, Bigler L, Gasser G, Schultz C, Wender PA, Jessen HJ. 2013. Cellular delivery and photochemical release of a caged inositol-pyrophosphate induces PH-domain translocation *in cellulo*. Nat Comm 7:10622. doi:10.1038/ncomms10622. PMC4743007 26842801

[30] Otwinowski Z, Minor W. 1997. Processing of X-ray diffraction data collected in oscillation mode. Methods Enzymol 276:307–326. doi:10.1016/S0076-6879(97)76066-X. 27754618

[31] Adams PD, Afonine PV, Bunkóczi G, Chen VB, Davis IW, Echols N, Headd JJ, Hung LW, Kapral GJ, Grosse-Kunstleve RW, McCoy AJ, Moriarty NW, Oeffner R, Read RJ, Richardson DC, Richardson JS, Terwilliger TC, Zwart PH. 2010. PHENIX: a comprehensive Python-based system for macromolecular structure solution. Acta Crystallogr D Biol Crystallogr D66:213–221. doi:10.1107/S0907444909052925. PMC2815670 20124702

[32] Kelley LA, Mezulis S, Yates CM, Wass MN, Sternberg JE. 2015. The Phyre2 web portal for protein modeling, prediction and analysis. Nat Protoc 10:845–858. doi:10.1038/nprot.2015.053. 25950237 PMC5298202

[33] Jones TA, Zou JY, Cowan SW, Kjeldgaard MA. 1991. Improved methods for building protein models in electron density maps and the location of errors in these models. Acta Crystallogr A Found Crystallogr 47:110–119. doi:10.1107/S0108767390010224. 2025413

