# Supplementary figures and images for: Structures of Fission Yeast Inositol Pyrophosphate Kinase Asp1 in Ligand-Free, Substrate-Bound, and Product-Bound States
Source: mBio. 2022 Dec 5;13(6):e03087-22. doi: 10.1128/mbio.03087-22 (PMC9765450; doi:10.1128/mbio.03087-22)

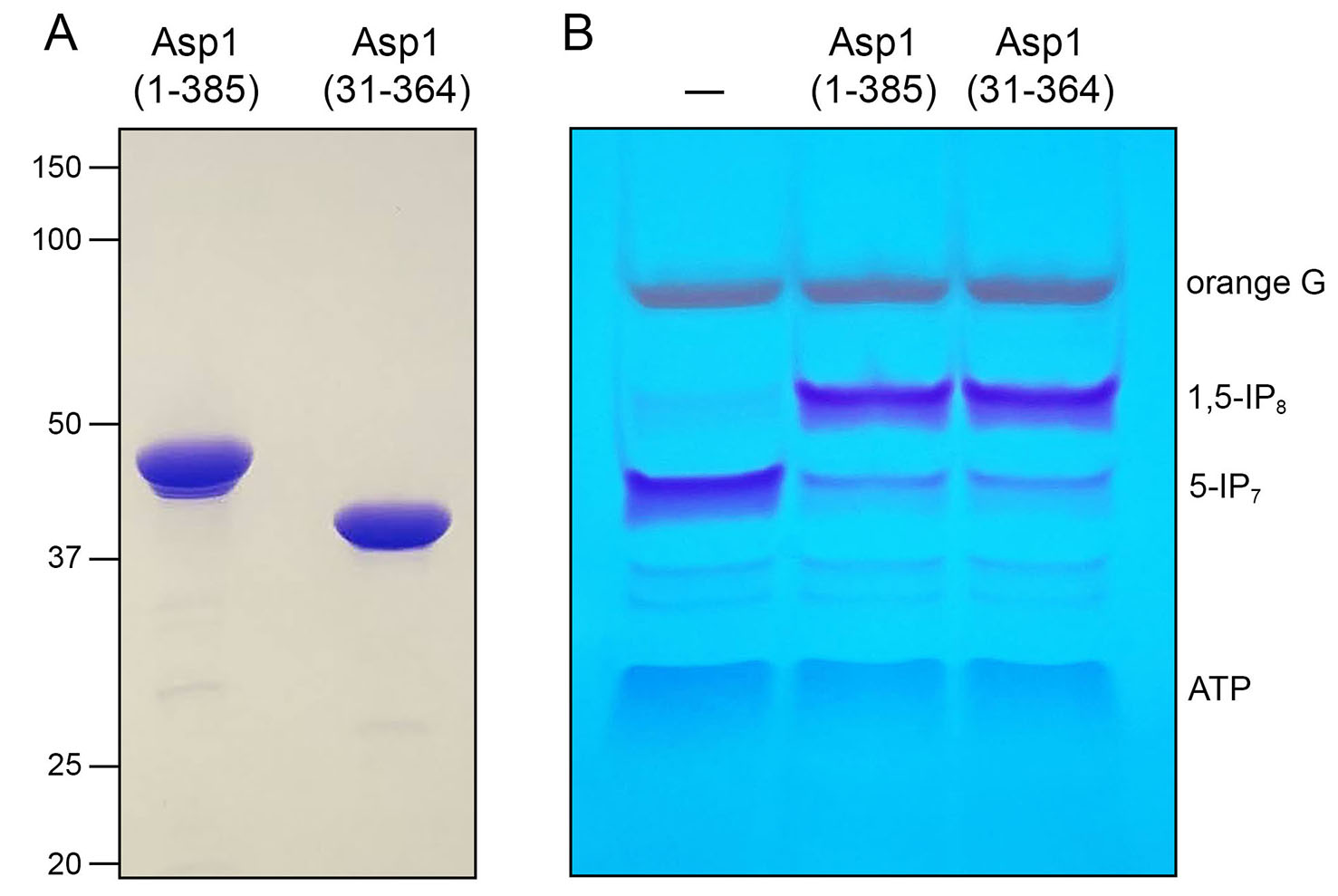

Supplement: FIG S1 [file mbio.03087-22-s0003.jpg]

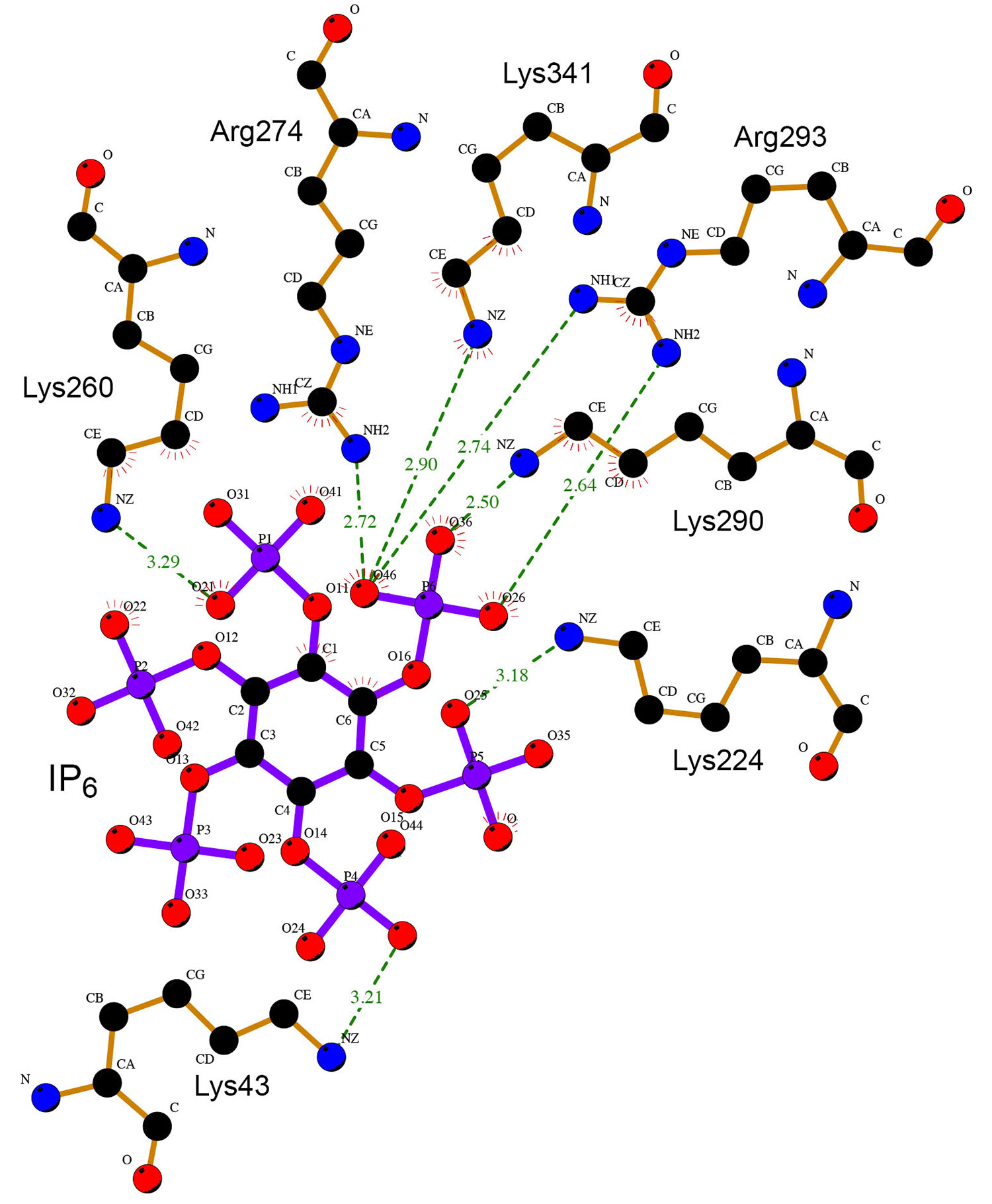

Supplement: FIG S2 [file mbio.03087-22-s0004.jpg]

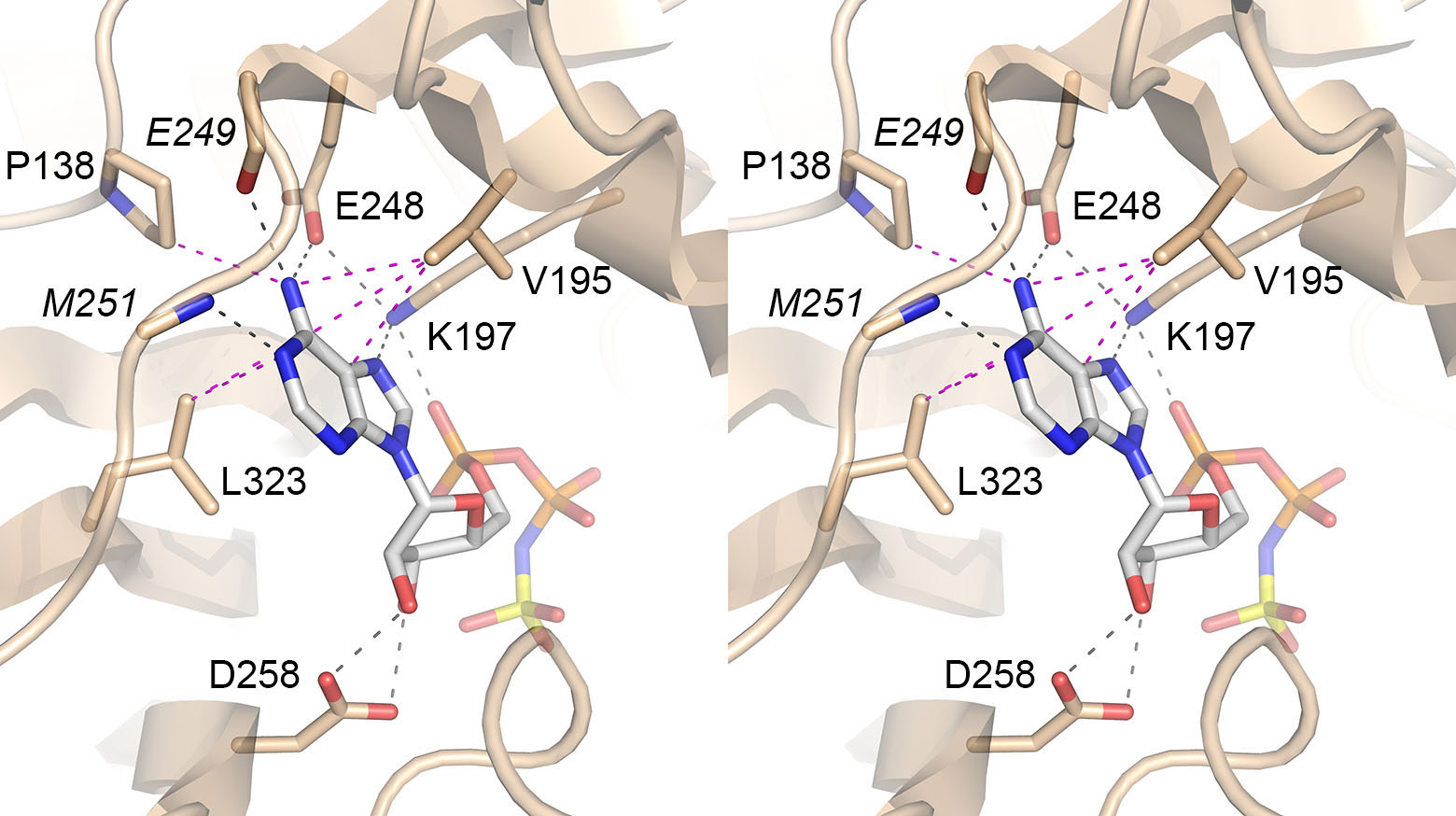

Supplement: FIG S3 [file mbio.03087-22-s0005.jpg]

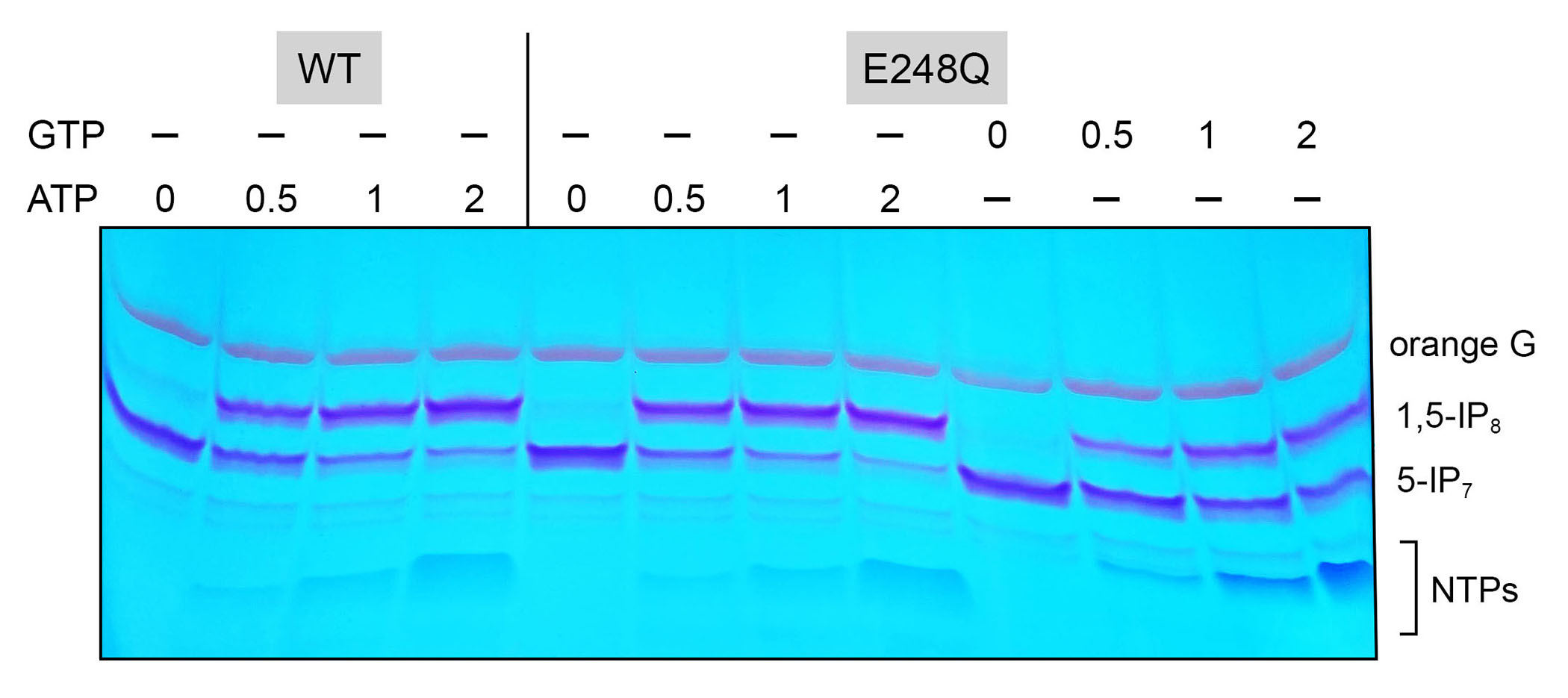

Supplement: FIG S4 [file mbio.03087-22-s0006.jpg]

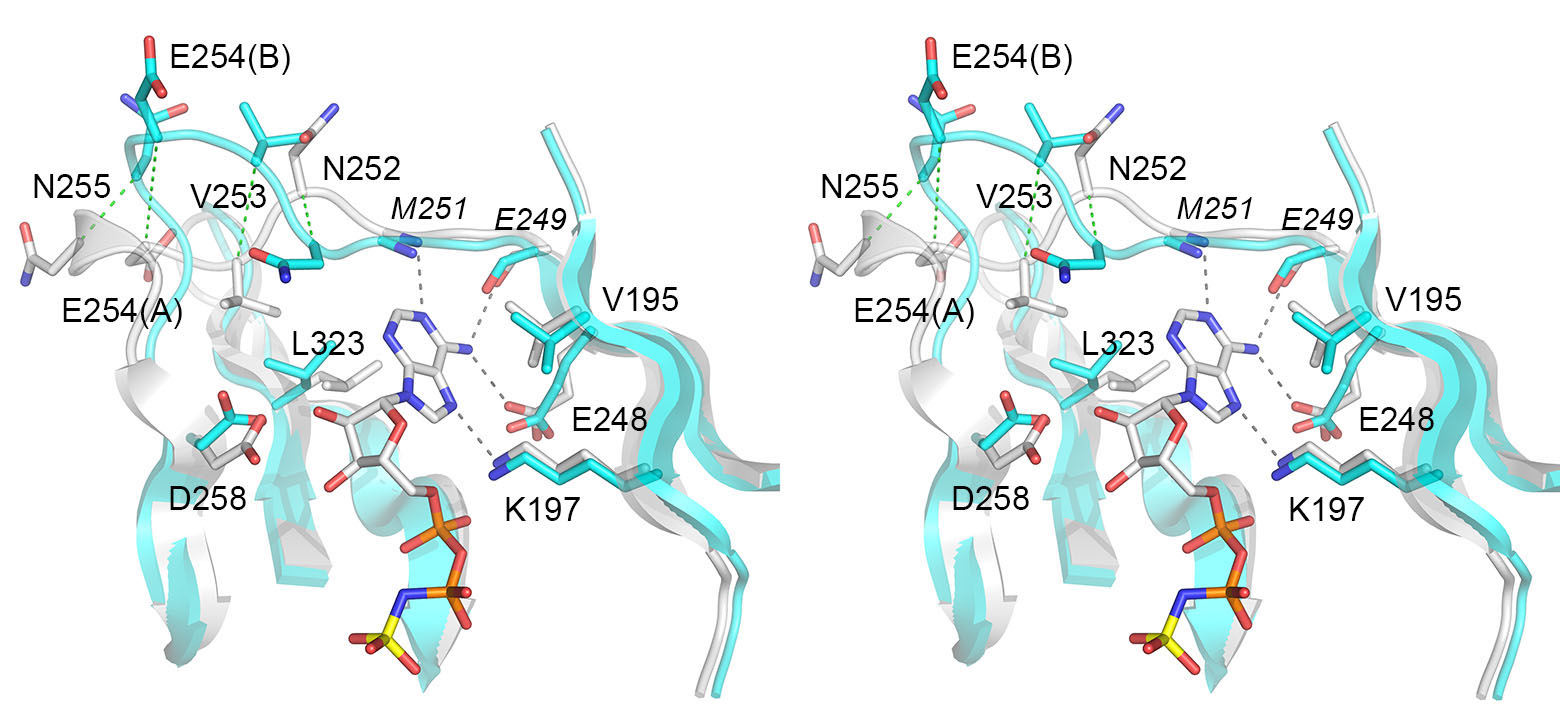

Supplement: FIG S5 [file mbio.03087-22-s0007.jpg]
