# Supplementary material for: Structures of Fission Yeast Inositol Pyrophosphate Kinase Asp1 in Ligand-Free, Substrate-Bound, and Product-Bound States
Source: mBio. 2022 Dec 5;13(6):e03087-22. doi: 10.1128/mbio.03087-22 (PMC9765450; doi:10.1128/mbio.03087-22)
Supplement: TABLE S1 [file mbio.03087-22-s0001.pdf]

Table S1. Crystallographic Data and Refinement Statistics

Asp1-(31-364)	WT	WT	WT	WT	WT	K47A-R50A
	ADPNP•Mg•IP_6	ADPNP•Mg•5-IP_7	ADPNP•Mn•IP_6	ADP•Mg•5-IP_7	1,5-IP_8	ATP•Mg•5-IP_7
**Data collection**						
Beamline	24ID-E	24ID-E	24ID-E	24ID-E	24ID-C	24ID-C
Space group	$P2_1$	$P2_1$	$P2_1$	$P2_1$	$P2_1$	$P2_1$
Cell dimensions<br>a, b, c (Å)<br>α, β, γ (°)	 47.56, 86.99, 86.29<br>90, 95.2, 90	 47.60, 87.51, 85.99<br>90, 94.8, 90	 48.08, 87.78, 86.05<br>90, 94.9, 90	 47.60, 87.12, 86.03<br>90, 94.7, 90	 47.86, 88.00, 86.18<br>90, 94.2, 90	 47.78, 88.50, 86.60<br>90, 94.8, 90
Resolution (Å)	50-1.9<br>(1.93-1.90)	50-1.7<br>(1.74-1.70)	50-1.7<br>(1.74-1.70)	50-1.9<br>(1.93-1.90)	50-1.6<br>(1.63-1.60)	50-2.0<br>(2.03-2.0)
Wavelength (Å)	0.9792	0.9792	0.9792	0.9792	0.9792	0.9792
R_{pim}	0.040 (0.334)	0.033 (0.278)	0.035 (0.380)	0.052 (0.346)	0.031 (0.333)	0.031 (0.401)
CC(1/2)	0.993 (0.543)	0.995 (0.784)	0.996 (0.601)	0.994 (0.804)	0.995 (0.732)	0.998 (0.697)
<I>/<σI>	26.6 (2.2)	29.1 (3.2)	31.3 (2.0)	25.2 (2.6)	38.3 (2.5)	32.2 (2.6)
Completeness (%)	98.7 (99.3)	95.9 (87.8)	97.7 (69.8)	95.2 (97.6)	98.0 (97.8)	98.6 (98.9)
Redundancy	4.0 (3.9)	3.9 (3.7)	4.2 (3.4)	2.9 (2.6)	4.2 (4.3)	4.3 (4.4)
Unique reflections	54255	72264	75427	52215	91920	48016
**Refinement**						
R_{work} / R_{free}	0.197 / 0.226	0.182 / 0.213	0.176 / 0.205	0.197 / 0.234	0.193 / 0.219	0.195 / 0.235
B-factors ($Å^2$)<br>Average/Wilson	 34.6 / 26.9	 25.7/ 18.8	 27.5 / 20.8	 36.7 / 29.6	 28.8 / 22.4	 39.2 / 31.8
RMS deviations<br>bond lengths (Å)<br>bond angles (°)	 0.010<br>1.15	 0.007<br>1.026	 0.008<br>1.09	 0.008<br>1.06	 0.008<br>1.06	 0.010<br>1.06
Ramachandran<br>% favored<br>% allowed<br>outliers	 97.6<br>2.4<br>0	 98.3<br>1.7<br>0	 98.8<br>1.2<br>0	 98.6<br>1.4<br>0	 98.7<br>1.3<br>0	 97.7<br>2.3<br>0
**Model contents**						
Protomers/ASU	2	2	2	2	2	2
Protein residues	640	647	647	647	641	646
Ions	1	1	2	3	0	2
Ligands	2	2	2	2	1	2
Water	460	661	725	416	710	419
**PDB ID**	8E1V	8E1T	8E1S	8E1H	8E1J	8E1I
