# Supplementary material for: Structures of Fission Yeast Inositol Pyrophosphate Kinase Asp1 in Ligand-Free, Substrate-Bound, and Product-Bound States
Source: mBio. 2022 Dec 5;13(6):e03087-22. doi: 10.1128/mbio.03087-22 (PMC9765450; doi:10.1128/mbio.03087-22)
Supplement: TABLE S2 [file mbio.03087-22-s0002.pdf]

Table S2: Structural Homologs of *S. pombe* Asp1 kinase

	pdb ID	Z score	% identity	root mean square deviation
Human PPIP5K2	3T9F	41.1	51	1.4 Å at 304 Cα positions
E. coli RimK L-glutamate ligase	5ZCT	20.9	14	2.8 Å at 255 Cα positions
Human ITPK1	2QB5	19.6	15	2.9 Å at 251 Cα positions
Entamoeba ITP5/6K	1Z2O	19.2	17	2.9 Å at 242 Cα positions
Streptomyces DcsG	6JIL	18.3	15	3.3 Å at 251 Cα positions
Rat synapsin I	1PK8	17.8	13	3.1 Å at 240 Cα positions
Plesiocystis PsnB	7DRM	17.1	14	3.2 Å at 239 Cα positions
Burkholderia D-Ala-Ala ligase	5NRI	17.1	12	3.4 Å at 257 Cα positions
